# Prospecting microbiota of Adriatic fish: *Bacillus velezensis* as a potential probiotic candidate

**DOI:** 10.1186/s42523-025-00429-5

**Published:** 2025-06-14

**Authors:** Jerko Hrabar, Ivana Babić, Slaven Jozić, Željka Trumbić, Adele Pioppi, Lasse Johan Dyrbye Nielsen, Ana Maravić, Tina Tomašević, Ákos T. Kovacs, Ivona Mladineo

**Affiliations:** 1 Institute of Oceanography and Fisheries, Split, Croatia; 2 Ruđer Bošković Institute, Zagreb, Croatia; 3 Department of Marine Studies, University of Split, Split, Croatia; 4 Institute of Biology, Leiden University, Leiden, The Netherlands; 5 DTU Bioengineering, Technical University of Denmark, Kgs Lyngby, Denmark; 6 Faculty of Science, University of Split, Split, Croatia; 7 Institute of Parasitology, BC CAS, Česke Budêjovice, Czech Republic; 8 Institute for Marine and Antarctic Studies, University of Tasmania, Hobart, TAS Australia

**Keywords:** Aquaculture, Probiotics, *Dicentrarchus labrax*, *Sparus aurata*, *Bacillus velezensis*, Whole genome sequencing

## Abstract

**Background:**

Aquaculture is one of the fastest growing sectors of food production and covers more than half of the market demand for fish and fishery products. However, aquaculture itself faces numerous challenges, such as infectious disease outbreaks, which are one of the limiting factors for the growth and environmental sustainability of modern aquaculture. Understanding the composition and diversity of the gut microbiota of fish is important to elucidate its role in host health and aquaculture management. In addition, the gut microbiota represents a valuable source of bacteria with probiotic potential for farmed fish.

**Results:**

In this study, we analysed the intestinal microbiota of two economically important fish species, the European seabass (*Dicentrarchus labrax*) and the gilthead seabream (*Sparus aurata*), using 16S rRNA gene amplicon sequencing. The taxonomic analysis identified 462 amplicon sequence variants at a similarity level of 99 and showed similar alpha diversity indices between seabass and gilthead seabream. Beta diversity analysis showed no significant differentiation in gut microbiota between fish species or aquaculture sites. Among the culturable isolates, a high proportion of *Photobacterium damselae* and *Bacillus* spp. was detected. We selected a single *Bacillus velezensis* isolate and further characterised its biosynthetic potential by performing whole genome sequencing. Its genome contains biosynthetic gene clusters for most of the common secondary metabolites typical of *B. velezensis*. Antibiotic susceptibility testing showed the sensitivity of the selected isolates to several antibiotics according to EFSA recommendations. Furthermore, stimulation of peripheral blood leukocytes (PBL) with *B. velezensis* resulted in a strong pro-inflammatory response, with a pronounced upregulation of cytokines *il1b*, *il6*, *tnfa* and *il10* observed over time.

**Conclusions:**

Overall, this study provides an insight into the composition of the intestinal microbiota and the diversity of culturable intestinal bacteria of two economically most important fish species from Adriatic cage culture and sheds light on the autochthonous intestinal *B. velezensis* as a promising probiotic candidate for Mediterranean aquaculture.

**Supplementary Information:**

The online version contains supplementary material available at 10.1186/s42523-025-00429-5.

## Introduction

Aquaculture is one of the crucial and the fastest growing food-supply sectors, with a global production of 59.4 million tonnes of finfish in 2024, of which around 2.1 million tonnes were produced in marine and coastal aquaculture facilities in Europe [1]. The most produced aquaculture fish species in Europe include gilthead seabream, *Sparus aurata* (GSB) and European seabass, *Dicentrarchus labrax* (ESB), whose production reached 245,402 tonnes and 256,577 tonnes respectively in 2022 [2], accounting for 95% of total finfish production in the Mediterranean. EU countries Greece, Italy, Spain and Croatia, together with non-EU countries, Turkey, Egypt and Tunisia, are traditionally the largest producers of both species, accounting for more than 90% of production [3]. However, conditions in rearing systems vary considerably between geographical regions and ultimately shape the etiological and epidemiological framework for fish health and welfare [4]. Infectious diseases cause significant economic losses to producers, estimated at several billion US dollars annually [5, 6]. Infectious disease outbreaks in aquaculture systems are one of the most important limiting factors in achieving the environmental sustainability of modern aquaculture [7]. This is aggravated by climate change, i.e. the rise in seawater temperature, particularly in the semi-enclosed Mediterranean, which is warming faster than other seas [8]. Studies suggest that increased local temperature can affect the physiology of bacterial cells and promote mutagenesis, leading to the emergence of antibiotic-resistant strains [9–11]. In addition to climate change, pollution of the marine environment by human activities (wastewater discharges, industrial effluents, agriculture, oil exploration and refining, etc.) also has a negative impact on the ability of farmed and wild fish populations to adapt to environmental changes and favours the invasion of bacterial and parasitic pathogens [12].

Relatedly, GSB and ESB aquaculture in the Mediterranean is burdened by a variety of infectious diseases that are caused by bacterial (aeromoniasis, mycobacteriosis, pseudotuberculosis, tenacibaculosis, vibriosis), viral (viral encephalopathy and retinopathy, lymphocystis), and parasitic pathogens (sparicotylosis) [13–16]. These infections, which are associated with higher fish mortality or the occurrence of severe epidemics, are likely to increase under the climate change scenario [13]. In addition, some of the fish pathogens causing these diseases are zoonotic, i.e. *Aeromonas hydrophila* (aeromoniasis) [17, 18], *Photobacterium damselae* subsp. *damselae* (pseudotuberculosis) [19] and *Mycobacterium* spp. (mycobacteriosis) [20, 21] and several *Vibrio* species, in particular *V. cholerae, V. vulnificus, V. parahaemolyticus, V. alginolyticus* and *V. harveyi* (reviewed by [22, 23]), showing an increasing global trend linked to anthropic activities.

Antimicrobial compounds are main solutions for the management of the bacterial diseases, but also major contributing factor in the spread of antimicrobial resistance (AMR) in many bacterial pathogens. Approximately 80% of antimicrobials used in aquaculture are estimated to be released into the environment with their activity intact, directly contributing to the increased risk of developing AMR [24–26] and raising public health concern.

One of the preventive measures for disease control is the use of probiotics in fish feed. Probiotics are useful, easy-to-use and genuine alternatives to the use of antibiotics. The WHO/FAO defines probiotics as live microorganisms that, when administered in appropriate doses, have a health benefit for the host [27]. The use of probiotics in aquaculture can increase nutrient utilisation and feed conversion and thus promote growth, disease resistance, rearing water quality and overall health of farmed fish by improving the internal microbial balance [28]. The latter is achieved through the production of various bacteriostatic or bactericidal compounds, including bacteriocins, siderophores, lysozymes, proteases, hydrogen peroxide and organic and volatile fatty acids, which can alter the pH value of the intestine [29]. It is also assumed that autochthonous probiotic strains offer better protection against resident pathogens, becoming predominant shortly after ingestion and remaining in the intestinal environment for a longer period [30]. The ability of probiotics to modulate the host immune system is of particular importance as they have been shown to improve the epithelial structure of the gut, modulate the secretion profiles of cytokines, influence the populations of T cells and increase antibody secretion [31].

To date, several yeast and bacterial strains have been investigated for their potential probiotic use in GSB and ESB aquaculture, either as live or heat-inactivated cells. In ESB, mostly lactobacilli were tested that exhibited multiple positive effects, such as the activation of various digestive enzymes [32], stimulation of the intestinal immune system of the larvae and downregulation of key pro-inflammatory cytokines [33], lowering of serum cortisol levels [34, 35], and promoting of weight gain by upregulating insulin-like growth factor 1 (*igf-1*) transcription [34]. In addition, several isolates of the genus *Bacillus* render positive effects in ESB on gut histology and microbial composition [36], increased disease resistance to *Vibrio anguillarum* [37–39], and increased feed digestibility [39]. In GSB, several other bacterial strains have been commonly tested as potential probiotics in addition to the lactic acid bacteria with proven positive effects on survival [40, 41], specific growth rate and digestive enzyme activities [41] or cellular innate immunity [42]. These include *Shewanella putrefaciens* (strain PdP11) that was shown to be beneficial in mitigating stress caused by high stocking density [43, 44], while several members of the genus *Bacillus* stimulated innate immunity [45, 46] and increased phagocytosis of *Edwardsiella tarda* and *V. anguillarum* [46].

Despite the progress made in use of probiotics in aquaculture, broadening the knowledge about the diversity, dynamics and functionality of the fish microbiome is required before favourable microbial strains can be selected and used as potential probiotics. Hence, the present study focused on two Adriatic cage-reared fish species, European seabass (*Dicentrarchus labrax*) and gilthead seabream (*Sparus aurata*), with the main objectives to: i) examine the taxonomic composition and diversity of dominant gut microbial community using the 16S rRNA gene metabarcoding amplicon analysis; ii) identify culturable bacterial isolates from the gut of both fish species; iii) characterise in silico the biosynthetic potential of a selected *Bacillus* isolate using whole genome sequencing; and iv) evaluate the immunomodulatory properties of the selected *Bacillus* spp. isolate on fish peripheral blood leukocytes in vitro.

## Methods

### Fish intestines sampling

**Fig. 1:**
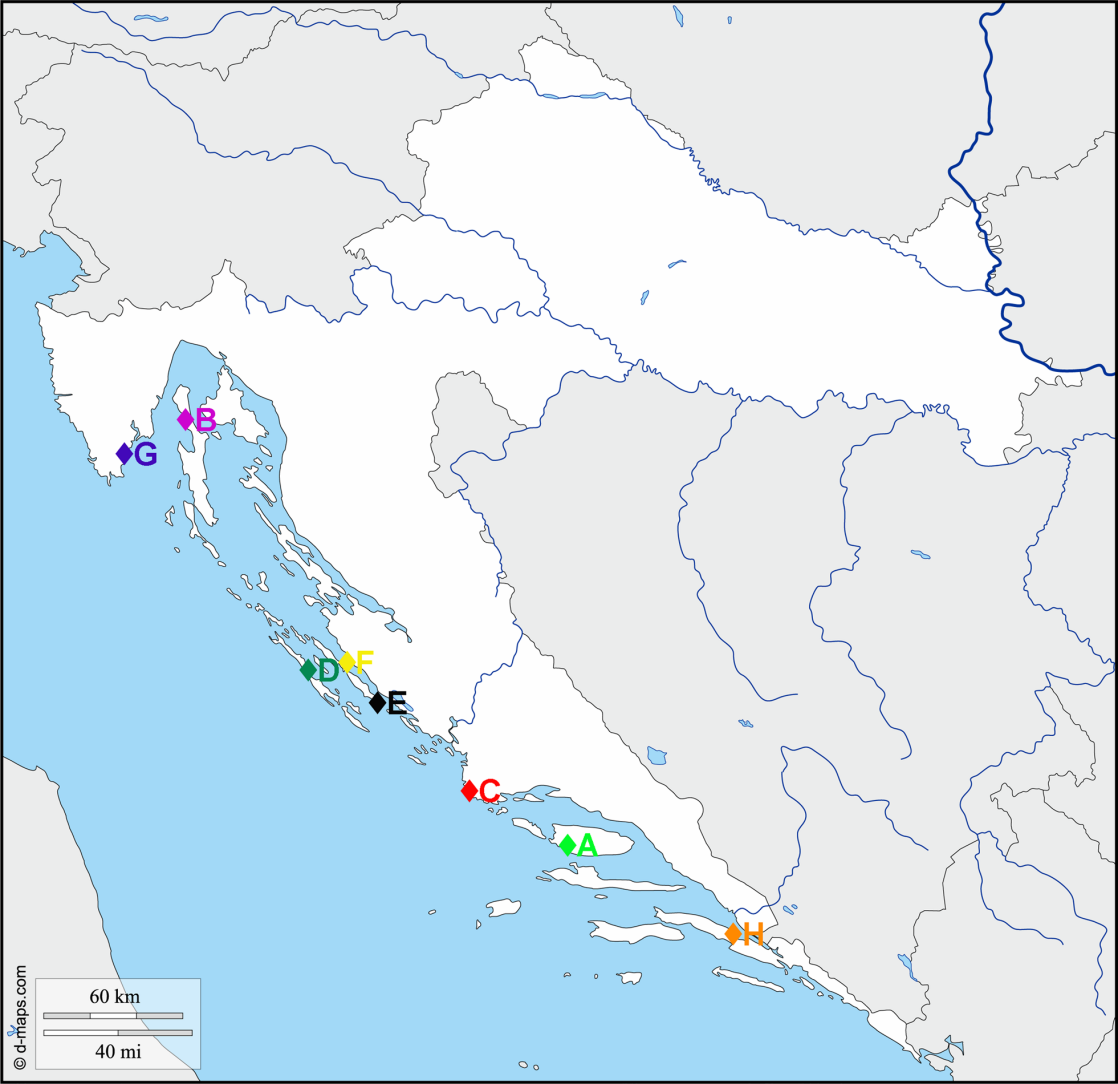
Map of Adriatic Sea with sampling locations. Shown are locations of farms from which European seabass (*Dicentrarchus labrax*) and gilthead seabream (*Sparus aurata*) were sampled in 2019. Note that from farm G only European seabass sample was collected. (Map downloaded from https://d-maps.com/carte.php?num_car=5352&lang=en)

Market-size European seabass (*Dicentrarchus labrax*, ESB) weighing 378.1 g ± 108 (mean ± SD) and measuring 33.9 cm ± 3.1 (mean ± SD) in length, and gilthead sea bream (*Sparus aurata*, GSB) weighing 381.3 g ± 85.2 and measuring 28.7 cm ± 2.5 in length were sampled from eight farms (Sites A-H) along the eastern Adriatic coast in June and July 2019 (Fig. 1). The exception was farm G, where only ESB was sampled. Five specimens of each species were sampled randomly from each farm, resulting in 35 and 40 specimens of ESB and GSB, respectively. In the grow-out phase the fish were fed twice daily ad libitum with commercial feed and withdrawn food 24 h before capture. The fish were netted from the cages, anesthetised and killed by a blow on the head. The fish from all farms were transported to the Institute of Oceanography and Fisheries (Split, Croatia) in ice slurry and processed immediately upon arrival. In no case did more than 8 h elapse between catching and processing the samples. During this time, the fish were constantly immersed in ice slurry to ensure minimal changes in the microbial communities.

Before dissection, the ventral surface of each fish was disinfected with 70% ethyl alcohol. The entire intestine from the pyloric caeca to the anal region was aseptically removed and washed several times with sterile PBS. The intestines of five specimens of each species from each farm were pooled as a single sample, diluted with 0.85% physiological saline (10% w/v, i.e. 10 g of tissue in 90 mL of saline) and homogenised in a sterile blender for total 60 s (4 × 15 s with 15 s pause). This approach was deployed to focus on isolation and identification of culturable bacteria to select the probiotic candidates from, while still yielding the most representative signature of overall intestinal microbiome. Previously, we observed that such an approach enables the cultivation of a diverse population of intestinal bacteria [4]. To ensure the capturing of intracellular or loosely adherent bacteria that may detach from the intestinal mucosa during sampling, and avoid potential contamination from the environment, whole intestine was homogenised. This also facilitated a consistent mucosal sampling, since mucosal scrapings can be subject to variation depending on the pressure applied by the operators. Steps described below were undertaken to ensure higher biomass of microbial DNA from samples of homogenised intestines that are abundant in host DNA.

### Fish gut bacterial community DNA extraction and sequencing

Prior to extraction of DNA from the intestinal microbiota, intestinal homogenates prepared as described above were lyophilized to ensure higher biomass of microbial DNA and used as starting material. Intestinal microbial DNA was extracted using the PureLink™ Microbiome DNA Purification Kit (Invitrogen, Waltham, MA, US) according to the manufacturer’s instructions with slight modifications: i) to ensure better tissue lysis, 4 µL of Proteinase K (Ambion, Carlsbad, CA, US) were added to the S1 Lysis buffer and incubated at 55 °C for one hour; ii) tissue was homogenised by bead beating in MagNA Lyser (Roche Diagnostics, Manheim, Germany) in two one-minute cycles at 6,000 oscillations, with a five-minute incubation on ice between cycles.

To explore composition of the microbial community in the gut of two fish species, the hypervariable regions V3-V4 of the bacterial 16S rRNA genes [47] were first amplified with the forward primer 341 F (5′-CCTACGGGNGGCWGCAG-3′) and the reverse primer 802R (5′-GACTACHVGGGTATCTAATCC-3′). An Illumina library was then constructed using 16S Nextera two-step PCR and sequenced on an Illumina MiSeq platform (PE250) using the MiSeq reagent kit v2 (2 × 250 bp paired-end) from Microsynth, Switzerland.

The obtained raw amplicon reads were filtered, trimmed and clustered into unique amplicon sequence variants (ASVs) using the software ‘Quantitative Insights Into Microbial Ecology 2’ (QIIME2), release 2022.8 [48]. Briefly, raw demultiplexed paired-end fastq files were imported into QIIME2 using a manifest file and then quality filtered, trimmed, dereplicated, denoised, merged and analysed for chimaeras to generate amplicon sequence variants (ASVs) using the DADA2 plugin [49]. Taxonomy was assigned employing the naïve Bayesian classifier method using the Silva 16S database (version 138 from September 28, 2020), clustered at 99% similarity using the QIIME2 feature classifier plugin. A phylogenetic tree was created using fasttree2 based on mafft alignment of the ASVs as implemented in the q2-phylogeny plugin [50]. After quality control and taxonomic assignment, sequences feature table, metadata and taxonomy table were imported into R Software v.4.0.3. [51] and analysed using the ‘*phyloseq*’ package [52]. Based on the generated taxonomy, the taxonomy table was filtered to exclude from the dataset ASVs assigned to the class *Chloroplast, Mitochondria* and *Unclassified* (Additional file 1). Raw sequence reads were deposited in European Nucleotide Archive (ENA) (https://www.ebi.ac.uk/ena) under project accession number PRJEB72876.

### Bacterial isolation and identification of culturable bacteria

The intestinal homogenates were decimally diluted ninefold with physiological saline (10% v/v, i.e. 1 part homogenate and 9 parts of diluent). An aliquot of 200 μL of each dilution was inoculated onto 160 mm Tryptic Soy Agar (TSA) plates (Biolife, Monza, Italy) containing 2% NaCl and incubated at 25 °C for 72 h. Plates with clearly demarcated bacterial colonies were selected and the size and appearance of the colonies were noted. Ten colonies were selected from each plate to encompass variations in morphology and colouration of distinctive colonies. If no differences were observed in morphology and colouration of colonies, then ten colonies were selected at random. Colonies were re-inoculated onto fresh TSA plates to obtain pure cultures, and finally grown overnight in Tryptic Soy Broth (TSB) containing 2% NaCl at 25 °C. Half of the overnight culture was used for molecular identification of the bacterial isolates, while the other half was stored in 20% glycerol at −80 °C as stock cultures.

For molecular identification, DNA was extracted from selected colonies (N = 150) using the DNeasy Blood and Tissue Kit (Qiagen, Hilden, Germany) with a modified protocol for Gram-negative bacteria according to the manufacturer’s instructions. A 1.4 kb portion of the 16S rRNA gene was amplified with 0.2 μM universal primers 27 F 5′-AGAGTTTGATYMTGGCTCAG-3′ and 1429R 5′-ACGGGCGGTGTGTRCAA-3′ [53]. The rest of the reaction mixture consisted of 2.5 mM MgCl_2_, 200 μM dNTPs, 5 U/μL HotStarTaq DNA polymerase (Qiagen, Hlden, Germany), 1 μL DNA template and MiliQ water to a final volume of 25 μL. Annealing temperature was set to 55 °C. The PCR products were checked in a 1% agarose gel and sent, along with negative PCR controls, to Macrogen Europe, Amsterdam (Netherlands) for commercial purification and sequencing. The sequences obtained were aligned using the Clustal W algorithm implemented in the MEGA X software [54]. After trimming the primer annealing sites, the sequences were compared with the sequences deposited in GenBank and the Ribosomal Database Project II (RDB database; https://rdp.cme.msu.edu/) using the BLAST tool [55] and Seqmatch, respectively. Taxonomic identification was based on > 99% similarities and generated list of taxa that served to select a probiotic candidate for further characterisation.

### Whole genome sequencing of *Bacillus velezensis*

Based on previous extensive data on the antimicrobial [56] and immunomodulatory potential of different *Bacillus* species and their secondary metabolites [38, 46, 57, 58], we selected a single *Bacillus* spp. isolate to further characterise its probiotic potential in Mediterranean aquaculture. The selected isolate was grown overnight in lysogeny broth (LB, Carl Roth, Germany; 10 g/L tryptone, 5 g/L yeast extract, and 5 g/L NaCl). Genomic DNA for Illumina and Nanopore sequencing was extracted using the GeneMatrix Bacterial and Yeast Genomic DNA Purification Kit (EURx, Gdansk, Poland) according to the manufacturer’s instructions. Paired-end library was prepared using the NEBNext Ultra II DNA Library Prep Kit for Illumina (New England Biolabs, Ipswich, MA, US). Genomic DNA was randomly fragmented to 350 bp, end-polished, A-tailed, ligated with an adapter and finally enriched by PCR. The paired-end reads were generated on an Illumina NovaSeq 600 with 2 × 150 bp reads. For Nanopore sequencing, a sequencing library was prepared using an SQK-RBK110.96 barcoding kit (Oxford Nanopore Technologies, Oxford, UK) according to the manufacturer’s instructions. The library was sequenced on a MinION platform with an R9.4.1 flow cell, with a 48-h sequencing cycle. Reads were live base called, demultiplexed and quality controlled in MinKNOW GUI v.4.1.22.

For the de novo assembly, the Illumina and Nanopore reads were quality- and adapter- trimmed in AdaptRemoval v.2.3.1 [59] and Porechop v.0.2.4 [60], respectively. The trimmed reads from both sequencing platforms were hybrid assembled using Unicycler v.0.4.8 [61]. The complete circular chromosome was analysed with Bandage v.0.8.1 [62] and BUSCO v.4.1.4 [63] to assess core gene content and with CheckM v.1.2.2 for completeness and contamination level [64]. Automatic annotation was performed using the NCBI Prokaryotic Genome Annotation Pipeline. The genome assembly has been deposited in GenBank under BioProject accession number PRJNA1196159. antiSMASH 5.0 software [65] was used to predict the biosynthetic gene clusters (BGCs).

### Antibiotic susceptibility testing

The minimum inhibitory concentrations (MICs) of the antibiotics against the selected *Bacillus* sp. were determined using the broth microdilution method according to the guidelines of the European Committee for Antimicrobial Susceptibility Testing [66]. A total of ten antibiotics were included in this study (all from Sigma-Aldrich, USA): eight antibiotics (vancomycin, gentamicin, chloramphenicol, erythromycin, clindamycin, kanamycin, streptomycin and tetracycline) recommended by the European Food Safety Authority (EFSA) [67] and two antibiotics (imipenem and meropenem) recommended by the European Committee on Antimicrobial Susceptibility Testing (EUCAST) [66]. The microdilution assays were performed in 96-well microtiter plates with two-fold serial dilutions of the active substances ranging from 32 to 0.03125 mg/L of their final concentration. Bacterial cells grown in Mueller–Hinton broth (Biolife, Monza, Italy) were then added to a final inoculum density of 5 × 10^5^ colony forming units/mL and incubated at 35 °C for 18 h. After incubation, the MIC was recorded as the lowest concentration of antibiotic that showed no visually detectable bacterial growth in the wells. *Staphylococcus aureus* ATCC 29213 was used as a quality control strain. The MIC tests were performed in triplicate.

### In vitro assay of fish peripheral blood leukocytes (PBLs) immunostimulation by *Bacillus velezensis* and quantification of target genes expression

Blood from four ESB anaesthetised with MS222 was aseptically collected from caudal vein and placed in EDTA coated BD Vacutainers (BD, Plymouth, UK). PBLs were prepared by hypotonic lysis of erythrocytes according to Attaya et al. [68]. In brief, 4 mL of whole blood of each ESB was mixed with 36 mL of ice-cold Mili-Q water for 20 s. Subsequently, 4 mL of chilled PBS (10x) (Sigma, UK) was added to restore the isotonicity of the medium. The suspension was then incubated on ice for 5–10 min and filtered through a 70 µm cell strainer (Greiner Bio One, UK). The PBLs were pelleted by centrifugation at 200 × g for 5 min and washed once with incomplete cell culture medium, Leibovitz L-15 (Sigma, UK) supplemented with 100 IU/mL penicillin, 100 µg/mL streptomycin (P/S) and 1% foetal bovine serum (FBS) (Sigma, UK). Trypan blue staining was used to count the PBLs in Neubauer chamber and ensure viability greater than 95%. Finally, PBLs were resuspended in complete cell culture medium (as above, except 10% FBS) and seeded in 6-well cell culture plates at 2 × 10^6^ cells in 3 mL medium. The PBLs of each fish were then stimulated with 10^7^ CFU/mL of *B. velezensis* or 25 µg/mL lipoteichoic acid (LTA) of *B. subtilis* (Sigma, Israel) for 3, 6 and 18 h, respectively. Upon treatment, cells were resuspended in 1 mL of TRIzol (Ambion, Carlsbad, CA, US) and stored at −80 °C until RNA extraction. LTA, being the surface-associated adhesion amphiphile from Gram-positive bacteria, served as a positive control for the in vitro assay. Non-stimulated PBLs in culture medium were used as control. In total, 36 samples of PBLs were collected for RNA isolation (four individual fish × two treatments, control × three time points).

Total RNA from PBLs was extracted using the TRIzol method according to the manufacturer’s instructions and dissolved in 20 µL of Mili-Q water (Merck Millipore, Billerica, CA, US). Prior to cDNA synthesis, RNA samples were treated with DNase I, RNase-free (ThermoFisher Scientific, Vilnius, Lithuania) to avoid amplification of residual genomic DNA. cDNA was synthesised from 500 ng of total extracted RNA using the PrimeScript 1 st Strand cDNA Synthesis Kit (Takara, Shiga, Japan) according to the manufacturer’s instructions. Expression of target genes (Table 1) was quantified by real-time PCR using the LightCycler 480 SYBR Green I Master (Roche Diagnostics, Manheim, Germany) with the following cycling conditions: pre-incubation for 5 min at 95 °C, 40 cycles for 10 s at 95 °C, 20 s at 60 °C/62 °C, 30 s at 72 °C, with melting curves recorded from 75 to 98 °C to assess the specificity of each reaction. Elongation factor 1 a (*ef1a*), actin beta (*actb*) and 18S rRNA (*rna18s*) were used as reference genes due to their stability after BestKeeper analysis [69]. Prior to real-time PCR, the cDNA template was diluted 1:10 with Mili-Q water and 2.5 μL of cDNA was used for each reaction. The web interface of Primer3 (v.4.10.) was used to generate specific primers for toll-like receptor 2 (*tlr2*), while the other primers were used from previous studies (Table 1).

**Table 1:** Oligonucleotide primers used for target gene expression analysis in European seabass (*Dicentrarchus labrax*). Indicated are primer sequences, annealing temperature for each primer pair, efficiency, product size and respective reference from which the sequences were retrieved

Locus/Accession	Primer sequence (5´ → 3´)	Annealing (°C)	Efficiency (%)	Product size (bp)	Reference
interleukin 1 beta (*il1b*)	CATGAGCGAGATGTGGAGATCCAAGAT CATTGTCAGTGGGTGGTGGGTAATC	62	97.6	73	[70]
interleukin 6 (*il6*)	CATGCCCTGAGAAGTCCA TTGAGAAGAGCTGTGTAAGTGA	62	90.5	79	[70]
tumor necrosis factor a (*tnfa*)	TCTACAGCCAGGCGTCGTTCAG CCGCACTTTCCTCTTCACCATCGT	60	91.1	57	[70]
interleukin 10 (*il10*)	CAGTGCTGTCGTTTTGTGGAGGGTTTC TCTCTGTGAAGTCTGCTCTGAGTTGCCTTA	60	98.5	77	[70]
toll-like receptor 2 (*tlr2*)	GGCTAGCTGTAATCCACCTGTCA CAGCTGTATGGGTTGTTGAGCAG	62	95.3	154	This study
elongation factor 1 a (*ef1a*)	CTGGTGTTGGTGAGTTCGAGG GGGGTTGTAGCCGATCTTCTTG	60	96.5	203	[4]
18S rRNA (*rna18s*)	CCAACGAGCTGCTGACC CCGTTACCCGTGGTCC	60	96.3	200	[4]
actin beta (*actb*)	TGCTGTCCCTGTATGCCTCTG GGCTGTGGTGGTGAAGGAGTAG	60	99.2	176	[4]

#### Statistical analysis

### Intestinal microbiota analyses

Overall statistical analyses and visualisations were performed with R Software v.4.0.3. Bacterial diversity (alpha diversity, calculated intra-sample) and structure (beta diversity) were analysed using the ‘*phyloseq*’ package [52] and results were visualised using the *‘ggplot2*’ package [71] in RStudio. For these analyses, the samples were subsampled to a minimum of 45,295 reads per sample. This threshold was set by the smallest read count of sample 12 – *Sparus aurata* from farm A (Fig. 1 and Additional file 1) leaving all 15 samples for the diversity analyses, as the generated rarefaction curve was saturated (Additional file 2). Alpha diversity analysis included observed number of ASVs, Shannon’s diversity and Pielou’s evenness, which were compared using Kruskal–Wallis non-parametric hypothesis test. To compare the gut microbiota among the two fish species (i.e. beta-diversity), a Weighted and Unweighted UniFrac principal coordinates analysis (PCoA) was performed using the packages ‘*phyloseq*’ and *‘ggplot2*’. Beta diversity was tested with Permutational multivariate analysis of variance (PERMANOVA) with 999 permutations (function ‘adonis2’ using the ‘*vegan*’ package). Based on the assigned microbial taxonomy data using the Silva database in QIIME2, the average relative abundance on phylum and genus levels was calculated for each sample and used for visualisation with the *‘ggplot2*’ package. A Venn diagram was used to determine shared and unique ASVs between two fish species by using the MicEco R library (https://github.com/Russel88/MicEco). Finally, microbial composition was further analysed with a focus on core microbes. Core microbiota analysis was conducted for each fish species using the core_members function from the R ‘*microbiome*’ package, applying default thresholds (detection threshold: 0%, prevalence threshold: 50%) [72]. A heatmap was generated using the “*pheatmap*” package, displaying hierarchical clustering of both samples and taxa. To assess statistical differences in the abundance of each taxon between fish species, a Wilcoxon rank-sum test was applied. P-values were computed using the wilcox.test function from base R and corrected for multiple testing using the Benjamini–Hochberg false discovery rate (FDR) method (p.adjust, method = ”fdr”). Only unadjusted and FDR-corrected *p*-values were reported in the results table.

### Gene expression analyses

Expression of all genes was calculated according to formula Efficiency^(−Ct)^ and target genes were normalised against the geometric mean of the three housekeeping genes (*ef1a, rna18s, actb*). Log2 transformation, scaling and exploratory data analysis via principal component analysis (PCA) were conducted using R software v.4.3.0 [51] with *prcomp* function. As it was determined that one LTA sample at 18 h heavily deviated from the rest of the group, it was removed from further analyses. Differential expression analysis of target genes was performed in R with the ‘*limma’* package [73, 74]. The group means parametrization approach was applied to construct design matrix taking into account time and treatment. Contrasts were used to compare each treatment against its time matched control, differences between treatments (BV *vs* LTA for each group), and time points (Additional file 3). Fold changes greater than 2 (log2FC ≥ 1) were considered biologically significant, i.e. a change in expression large enough to manifest a specific effect. Statistical significance was set at Benjamini-Hochberg adjusted *p*-value < 0.05. All visualisations for qPCR analyses were performed using the package ‘*ggplot2*’[71] for R.

## Results

### Fish intestinal microbiota composition and diversity

To assess the bacterial diversity of GSB and ESB microbiomes, 15 samples were collected and analysed using 16S rRNA gene amplicon sequencing. A total of 1,428,925 raw reads were obtained from 15 samples included in the study. After the DADA2 processing [49] and filtering of the resulting table, 1,010,886 merged reads were obtained from 15 samples and a total of 462 ASVs were identified. Finaly, after taxonomy filtering by ‘*phyloseq*’ package in R, total of 949,486 reads from 15 samples and 427 ASVs remained. Additional file 1 lists the final number of obtained reads for each sample.

### Microbial diversity of fish-associated sample groups and the aquaculture location

#### Taxonomic richness (alpha and beta diversity)

**Fig. 2:**
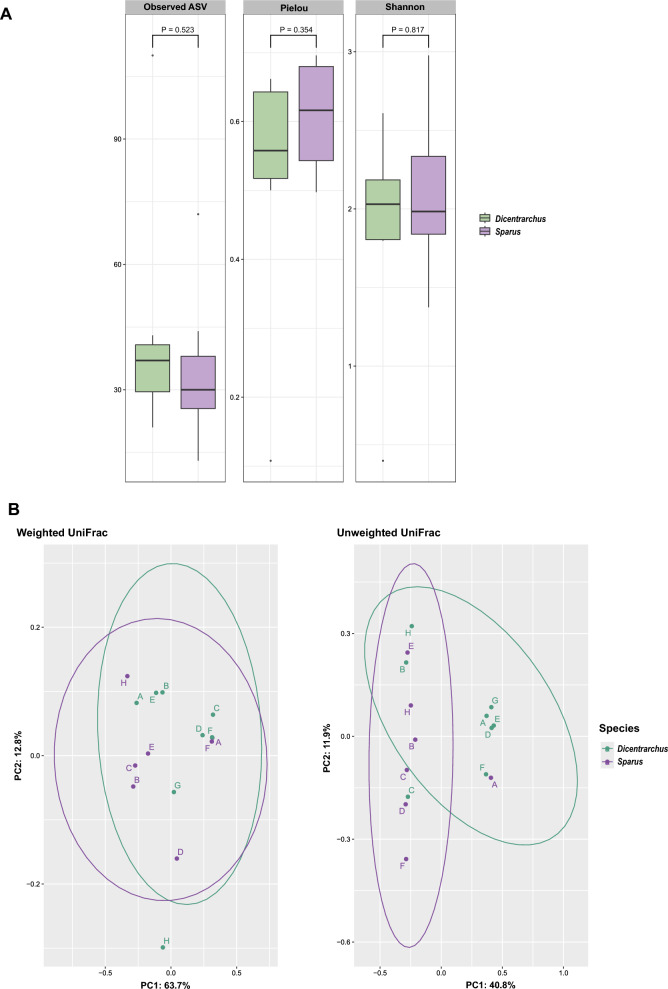
Alpha and beta diversity plots. **(A**) Alpha diversity analyses of gut microbiomes in two aquaculture fish species: *Dicentrarchus labrax* (green) and *Sparus aurata* (purple): Observed number of ASVs, Pielou’s evenness index and Shannon’s diversity index. Kruskal–Wallis hypothesis test P values are indicated above horizontal bars. (**B**) Principal coordinate analysis (PcoA) based on weighted and unweighted Unifrac distances of fish gut samples according to species and the locations. Fish farm location (A – H) match the labels on Fig. 1

The alpha diversity indices—the taxonomic richness index (observed number of ASVs), the Shannon’s diversity index and the Pielou’s evenness index—between two aquaculture fish species are represented in Fig. 2A. The ESB samples had a slightly higher number of observed ASVs overall compared to the GSB samples (i.e. 43 ASVs vs 35 ASVs on average), but no significant difference was found between the two (Kruskal–Wallis test: *P* = 0.523). According to Shannon’s diversity and Pielou’s evenness index, both species had similar species richness and evenness of the gut microbial community, with a mean of 1.9 and 0.52 for ESB and 2.09 and 0.6 for GSB, respectively, with no significant difference found between the two for each index (Kruskal–Wallis test: Shannon’s diversity—*P* = 0.817 and Pielou’s evenness—*P* = 0.354). List of values for the three alpha diversity indices for each sample is presented in the Additional file 4.

Beta diversity plots (Unweighted and Weighted UniFrac principal coordinates analysis (PCoA)) showed that overall there are no differentiation of fish gut samples according to the fish species (permutational multivariate analysis of variance [PERMANOVA]: *P* = 0.07, pseudo-F = 2.17, and *P* = 0.41, pseudo-F = 0.87, respectively) (Fig. 2B).

#### Microbial composition

**Fig. 3:**
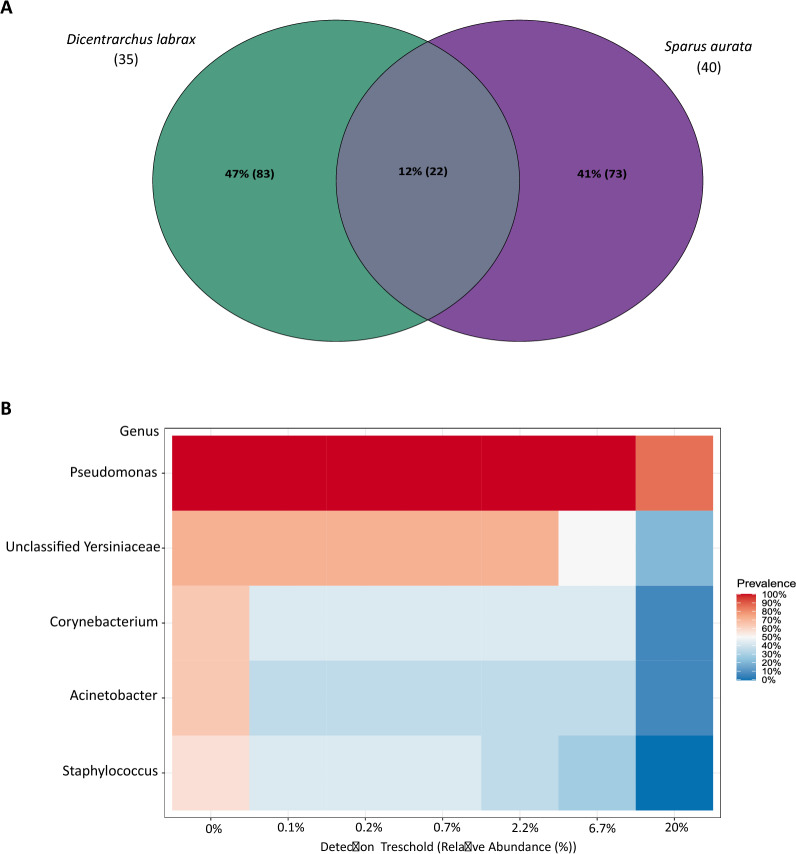
(**A**) Venn diagram displays the number of shared and unique ASVs among two fish species. (**B**) Heatmap of core microbiome at Genus level. Scale represents prevalence (relative abundance)

Venn diagram was constructed to identify the shared or special ASVs in two different fish species of intestinal samples. Interestingly, as shown in Fig. 3A, both sample groups had high percentage (47% ESB, 41% GSB) of unique ASVs, while shared only 12% (22) of similar number of ASVs.

**Fig. 4:**
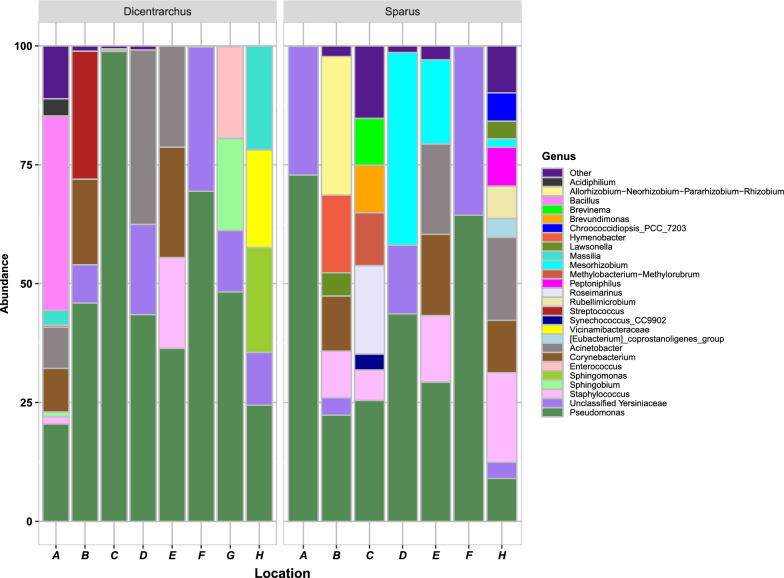
Taxonomical structure of bacterial assemblages with over 3% relative abundance. Taxa with < 3% are denoted as Other. Hypervariable V3-V4 regions of 16S rRNA were used to assign taxonomy at genus level in two aquaculture fish species – *Dicentrarchus labrax* and *Sparus aurata* at different locations (A – H) in the eastern Adriatic Sea. Fish farm location (A – H) match the labels on Fig. 1

Taxonomic analysis revealed 13 phyla and 58 genera in addition to an unclassified bacterial group, however only 9 phyla were abundant with more than 0.5% and 26 genera were abundant with more than 3% (Additional file 5 and Fig. 4). Two aquaculture fish species had gut microbiomes with a similar composition of core taxa (Fig. 3B and Additional file 5). *Pseudomonadota* (ex: *Proteobacteria*), *Bacillota* (ex: *Firmicutes*) and *Actinomycetota* (ex: *Actinobacteria*) were the core bacterial phyla in all samples and all three phyla were dominant > 70% (Additional file 5). Of the 10 most abundant phyla (Additional file 5) present in both species, *Pseudomonadota* were the most represented (ESB: 40.98–99.78%; GSB: 42.16–99.93%), followed by *Bacillota* (ESB: 0–45.48%; GSB: 0–32.68%) and *Actinomycetota* (ESB: 0–23.2%; GSB: 0.02–17.08%). In addition, the presence of the following taxa was detected in the gut microbiome of some ESB samples: *Acidobacteriota* (location H – 20.53%), *Deinococcota* (location A – 2.1%). The presence of the following taxa was detected in the gut microbiome of some samples of GSB: *Bacteroidota* (samples B – 16.33% and C – 18.63%), *Spirochaetota* (sample C – 9.87%) and Cyanobacteria (samples C – 3.33% and H – 7.53%).

*Pseudomonas*, Unclassified *Yersiniaceae, Corynebacterium, Acinetobacter* and *Staphylococcus* were present in all gut samples, making them the core bacterial genera (Fig. 3B). Moreover, *Pseudomonas* was the predominant genus in all samples of both species (ESB: 20.48–98.84%; GSB: 9.04–72.84%) (Fig. 3 and 4). Other four core genera were present in both species with the abundance > 3%, but only in some samples (Fig. 4): Unclassified *Yersiniaceae* (ESB: locations B – 8.04%, D – 18.95%, F – 30.32%, G – 12.89%, and H – 11.06%; GSB: locations A – 27.08%, B – 3.64%, D – 14.42, F – 35.39, and H – 3.83%), *Corynebacterium* (ESB: locations A – 9.17%, B – 17.96%, E – 23.2%; GSB: locations B – 11.55%, C – 6.41, E – 17.05% and H – 11.02%), *Acinetobacter* (ESB: locations A – 8.63%, D – 36.7% and E – 21.29%; GSB: locations E – 18.98% H – 17.42%), *Staphylococcus* (ESB: locations A – 1.52%, E – 19.07%; GSB: locations B – 9.83%, C – 6.41, E – 13.99% and H – 18.83%). The presence of 8 genera was detected in the gut microbiome of certain samples of ESB: *Acidiphilium* (location A – 3.62%), *Bacillus* (location A – 40.86%), *Enterococcus* (location G – 19.3%), *Massilia* (locations A – 3.13% and H – 21.87%), *Sphingobium* (location G – 19.31%), *Sphingomonas* (location H – 22.04%), *Streptococcus* (locations A – 0.39% and B – 26.92%), and *Vicinamibacteraceae* (location H – 20.53%). Similarly, the gut microbiome of 11 samples of GSB revealed the presence of certain genera that were absent in the ESB samples: [Eubacterium]_coprostanoligenes_group (location H – 3.95%), *Allorhizobium-Neorhizobium-Pararhizobium-Rhizobium* (location B – 29.15%), *Brevinema* (location C – 9.87%), *Brevundimonas* (location C – 10.05%), *Chroococcidiopsis*_PCC_7203 (location H – 5.99%), *Hymenobacter* (location B – 16.33%), *Lawsonella* (locations B – 4.89% and H – 3.7%), *Mesorhizobium* (locations C – 11.08%, D – 40.51%, E – 17.72%, H – 1.78%), *Peptoniphilus* (location H – 8.17%), *Roseimarinus* (location C – 18.64%), *Rubellimicrobium* (location H – 6.81%), and *Synechococcus_CC9902s* (location A – 3.32%).

**Fig. 5:**
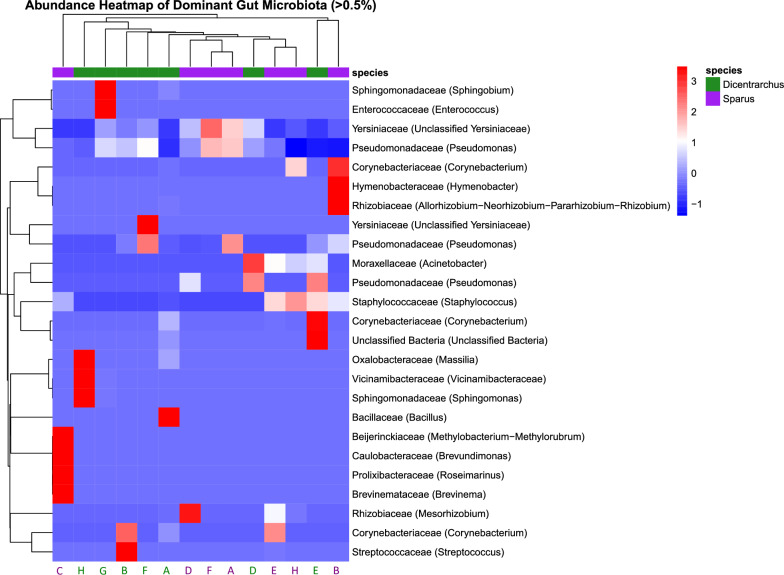
Abundance heatmap of discriminative gut microbiomes detected between two fish species – ESB (green) and GSB (purple). Taxonomic annotation was included at the family (Genus) level for labelling. A color-coded annotation bar was added above the heatmap to indicate host species identity, as well indicated in the fish farm location (A – H) that match the labels on Fig. 1

To investigate the dominant members of the gut microbiome in two aquaculture fish species (ESB and GSB), a heatmap was constructed using OTUs with relative abundances above 0.5% across samples (Fig. 5). Z-transformed abundances revealed distinct clustering of samples primarily driven by host species. Among the 27 dominant taxa visualized, statistical comparison using the Wilcoxon rank-sum test identified no significantly different taxa between the two fish species after FDR correction (FDR-adjusted *p* > 0.05 for all comparisons, Additional file 6). *Corynebacterium* and *Streptococcus* were more abundant in ESB, while *Pseudomonas* and *Yersiniaceae* (Unclassified) appeared more prevalent in GSB individuals. *Acinetobacter*, *Enterococcus*, and *Staphylococcus* were detected across both species without clear host specificity.

### Identification of cultivable bacteria

A total of 150 strains were isolated on TSA plates from fish intestine tissue. Of these, seven sequences obtained were of poor quality, and were excluded from further analyses. Comparison of the sequences with those deposited in the GenBank and RDP databases identified *P. damselae* as the most abundant cultivable bacterial species from the guts of both ESB and GSB, accounting for almost a third of all isolates identified (42/143, 29.37%). Other bacteria represented with more than five colonies cultivated from the guts of both ESB and GSB were *Bacillus* spp. (34/143, 23.77%), *Vibrio* spp. (19/143, 13.28%), *Staphylococcus* spp. (15/143, 10.49%) and *Psychrobacter* spp. (6/143, 4.19%). Most of the remaining bacteria were present with a single cultured colony (Additional file 7).

Assessing the prevalence of cultured isolates by farm and host species, the highest divergence with 6 different isolates from ten selected colonies was observed in ESB on farm A. The second highest divergence with five different isolates was observed in both ESB and GSB on farm B, GSB on farm D and in ESB from farm F. Finally, the lowest divergence with only *P. damselae* identified among the selected colonies was observed in ESB on farm E, and divergence with only *Bacillus* spp. was identified in GSB on farms E and H (Additional file 7).

### Whole genome sequencing of *Bacillus velezensis*

The genome sequence of the selected isolate, DL_A4 was determined using Illumina and Nanopore sequencing followed by de novo hybrid assembly [75, 76]. Based on the analysis of the Genome Taxonomy Database, the selected *Bacillus* spp. isolate was identified as *B. velezensis*, which was further supported by the analysis of the biosynthetic gene clusters (BGCs). The antiSMASH analysis identified the BGCs for most of the common specific secondary metabolites of *B. velezensis*, including surfactin, bacillaene, macrolactin, fengycin, difficidin, bacillibactin, and bacilysin. The biosynthetic potential for iturin is also present, “region 8” comprises two BGCs, namely the gene cluster for the synthesis of fengycin and iturin. While antiSMASH is unable to separate the two BGCs due to the overlapping genes, the presence of both BGCs is apparent.

### Antibiotic susceptibility testing

Next, MICs of selected antibiotics were tested against the *B. velezensis* isolate (Table 2). When both the EFSA [67] and the EUCAST [66] breakpoints values were applied for the MICs, the *B. velezensis* isolate was found to be sensitive to all ten antimicrobial agents.

**Table 2:** Antibiotic susceptibility phenotype of *B. velezensis* isolate (S = sensitive)

Antibiotic	MIC (mg/L)	Breakpoint (mg/L)	
		EFSA	EUCAST
vancomycin	0.25	S ≤ 4	S ≤ 2
gentamicin	0.125	S ≤ 4	–
kanamycin	0.5	S ≤ 8	–
streptomycin	4	S ≤ 8	–
erythromycin	0.125	S ≤ 4	S ≤ 0.5
clindamycin	0.25	S ≤ 4	S ≤ 1
tetracycline	8	S ≤ 8	–
chloramphenicol	2	S ≤ 8	–
imipenem	0.0625	–	S ≤ 0.5
meropenem	0.25	–	S ≤ 0.25

### Quantification of target genes expression in *B. velezensis*-stimulated fish peripheral blood leukocytes (PBLs)

**Fig. 6:**
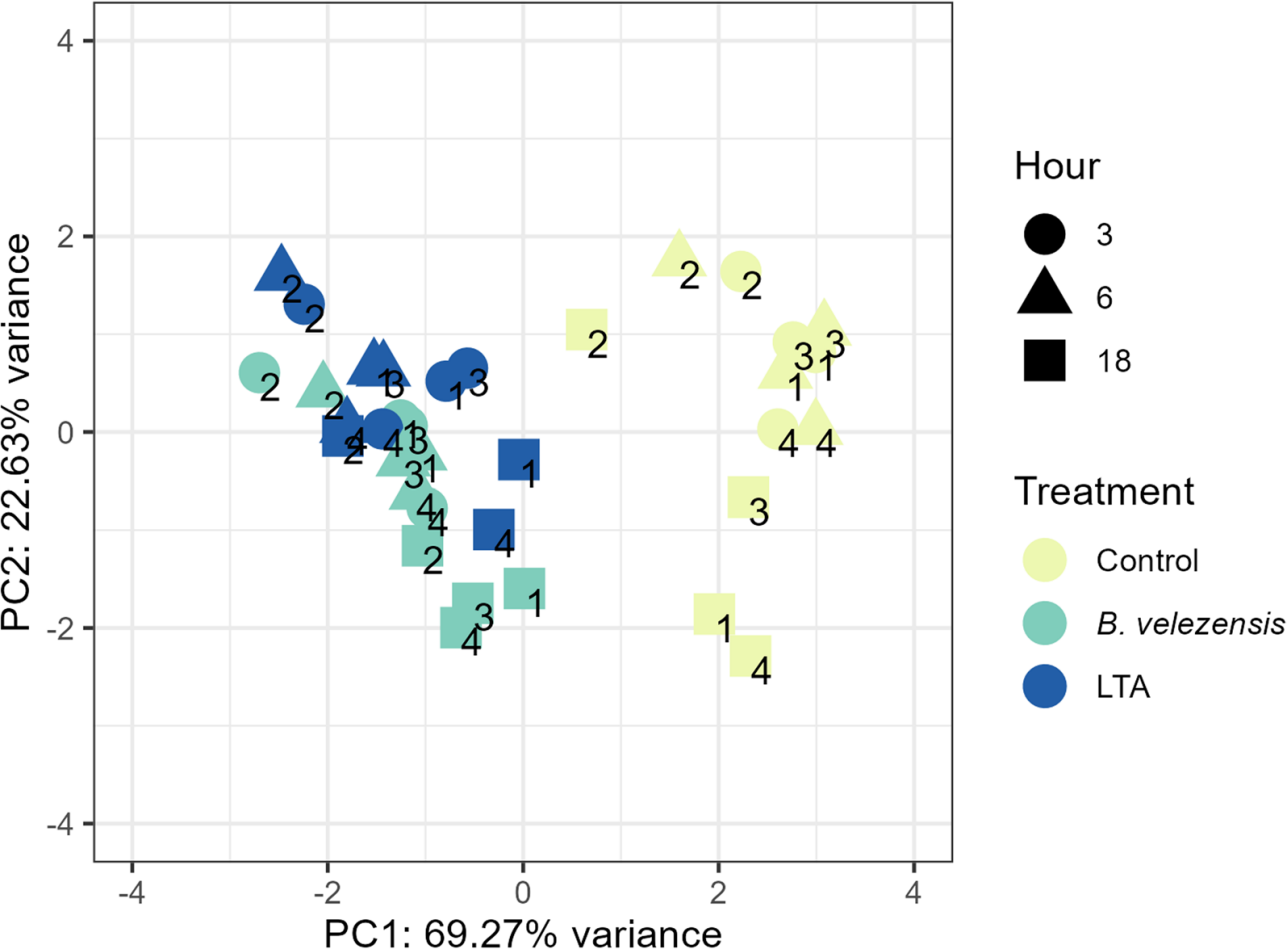
Principal components analysis for gene expression profiles during in vitro stimulation of European seabass PBLs. Relative positions of treatment groups (time-points and stimuli) are shown. Values are log2-transformed and scaled. Numbers within symbols indicate the fish PBLs were isolated from

PCA analysis was performed to determine similarities and differences in the expression of target genes analysed in fish peripheral blood leukocytes (PBLs) stimulated with live *B. velezensis* (BV) and lipoteichoic acid (LTA) for 3, 6 and 18 h (Fig. 6). PCA confirmed a clear separation of stimulated PBLs from non-stimulated controls. Stimulated PBLs formed a homogeneous cluster, whereas control PBLs were less homogeneous, mainly due to the variability observed 18 h after stimulation.

**Fig. 7:**
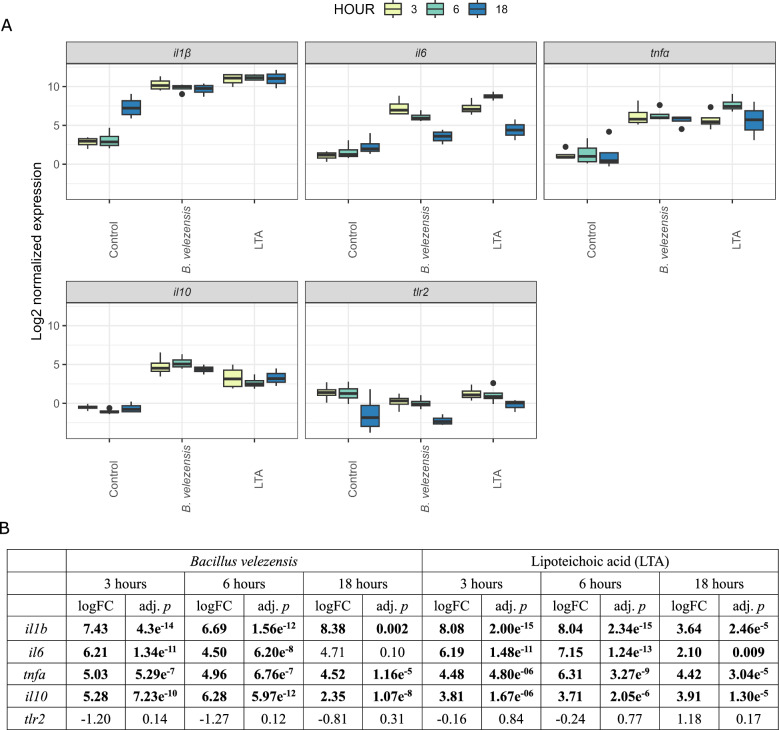
Expression of target genes in European seabass PBLs stimulated with *B. velezensis* and lipoteichoic acid (LTA). (**A**) Distribution of target gene expression is shown according to state (control, *B. velezensis*-stimulated and LTA-stimulated) and time. Box plots represent distribution of log2-normalised expression; **(B**) Log2-transformed fold changes of target genes expression in respect to time-matched controls with respective adjusted p-values. Statistically significant fold changes are indicated in bold

A strong pro-inflammatory response was observed as early as 3 h after stimulation, which slowly decreased until 18 h after stimulation (Fig. 7A). Nevertheless, the expression of all genes, except for toll-like receptor 2, remained high throughout the experiment. Of the five target genes, only *tlr2* was not differentially expressed in both treatments and at all time points. In fact, *tlr2* was constantly downregulated compared to time-matched controls, except for LTA stimulation at 18 h when induction of *tlr2* was observed. Although a biologically significant change in the expression of *tlr2* was detected in BV-stimulated PBLs 3 and 6 h after stimulation and in LTA-stimulated PBLs 18 h after stimulation (log2FC > 1) (Fig. 7B), this change in expression was not statistically significant compared to the time-matched non-stimulated control (*p* > 0.05). The highest average expression was measured for *il1b*, while the lowest average expression was measured for *il10*. The highest fold-change (FC) was observed for *il1b* in LTA-stimulated PBLs 3 and 6 h after stimulation and in BV-stimulated PBLs 3 h after stimulation, respectively, while the lowest significant FC was measured for *il6* in BV- and LTA-stimulated PBLs 18 h after stimulation (Fig. 7B).

When comparing the expression of target genes between treatments, both BV- and LTA-stimulated PBLs generally responded in the same way. The only statistically significant difference between treatments was observed for *il6* and *il10* 6 h after stimulation (Additional file 3). While *il6* was more strongly expressed in LTA-stimulated PBLs, BV-stimulated PBLs expressed more *il10* 6 h after stimulation. Assessing the differences in expression of the target genes between the time points, significant differences in expression were only observed for *il1b* and *il6* between 6 and 18 h post infection for both treatments (Additional file 3).

## Discussion

Probiotics in aquaculture have various beneficial aspects such as the potential to improve the immune status and performance of farmed fish. Isolation of autochthonous candidates is promising for successful colonisation of the gastrointestinal tract of farmed fish as they can be easily adapted to other contemporary farmed fish species [77]. In addition, the use of probiotics represents an effective strategy to combat pathogens through a variety of mechanisms as an alternative to antibiotic treatment [78]. The main objective of this study was to characterise the diversity of gut microbial communities and to investigate the probiotic potential of autochthonous gut bacteria of Adriatic cage-reared gilthead seabream, *Sparus aurata* (GSB) and European seabass, *Dicentrarchus labrax* (ESB) for possible use as a feed additive in mariculture. A single isolate of *Bacillus* spp. was selected and further characterised for its biosynthetic and immunostimulatory potential by WGS and in vitro stimulation of fish PBLs.

First, we characterised the composition of the gut microbiota of both ESB and GSB from eight aquaculture sites both by high-throughput Illumina 16S rRNA gene amplicon sequencing and by culture. The patterns of gut microbial communities and the identification of the dominant microbiome in the fish gut are fundamental for improving the physiological performance of the host. From analysing the taxonomic composition at the phylum level, it can be observed that *Pseudomonadota* (ex-*Proteobacteria*), *Bacillota* (ex-*Firmicutes*) and *Actinomycetota* (ex-*Actinobacteria*) were the most dominant phyla with similar relative contribution in samples of both fish species. *Pseudomonadota* and *Bacillota* are typically dominant bacteria associated with fish in both marine and freshwater habitats [79–83]. *Pseudomonadota* is the most abundant phylum in the healthy intestine and its disbalance may reflect a possible microbial signature of dysbiosis and disease in the gut microbiota [84]. Its predominance in both fish species is indicative of its importance for the host and ubiquitous presence in the environment. *Bacillota* are shown to be abundant in the gut of European seabass fed with plant-based diet [83]. *Actinomycetota* play the main role in maintaining gut homeostasis and are widely used as probiotics [85]. At the genus level, *Pseudomonas* (*Pseudomonadota*) was the predominant genus in the gut samples of both fish species, together with the Unclassified *Yersiniaceae* (*Pseudomonadota*), genus that was present in 10 samples (5 ESB and 5 GSB). The genus *Pseudomonas* can typically be found in the gut microbiota of other fish and is known to have probiotic properties [86–88]. *Yersiniaceae* (*Pseudomonadota*) are one of the seven families belonging to the order *Enterobacteriales* that contains common fish pathogens that can cause mild to severe fish diseases [89].

Previous studies indicated that the host species is the main factor influencing variations in the diversity and structure of the fish gut microbiota [88, 90–92]. Here, with respect to α-diversity indices (observed number of ASVs, Pielou’s evenness, Shannon index) the gut microbiota of the ESB and GSB had similar values. Regarding the PCoA plot, both weighted and unweighted analyses indicated that the microbiota of the ESB and GSB overlap and do not differ significantly from each other. These results could be related to the similar diet in the same aquaculture farms. Lower number of ASVs observed could be due to the method we used to obtain the intestinal homogenates for growing the culturable bacteria. We homogenised the whole gut tissue instead of using only the mucosal scrapings, which may have resulted in a lower diversity of the gut microbiome due to excessive dilution with host DNA or degradation of microbial DNA. However, as a potential probiotic should be easy to grow in culture, we opted for this approach to obtain the culturable isolates, especially as it proved satisfactory for obtaining a diverse population of culturable bacteria in our earlier study [4].

A high proportion (> 80%) of the cultivable bacterial isolates belonged to only five taxa, namely *P. damselae*, *Bacillus* spp., *Vibrio* spp., *Staphylococcus* spp. and *Psychrobater* spp. Although a rich medium was used to culture the bacteria, namely tryptic soy broth/agar, it is possible that the culture conditions did not favour the growth of some of the fastidious bacteria, found in the fish gut, so that only a few taxa predominated in the grown cultures.

A surprising finding was that almost 30% of the colonies grown belonged to *P. damselae*. The bacterium comprises two categories of subspecies, *P. damselae* subsp. *damselae* and *P. damselae* subsp. *piscicida* [93]. Unlike the culture method, by the 16S rRNA gene amplicon sequencing the presence of *P. damselae* was detected only in GSB sample from farm location C in total of < 3%, possibly due to the sampling method used to obtain the intestinal homogenates or culture conditions that favoured only some bacteria, as already described. Biases intrinsic to amplicon sequencing, such as primer amplification bias or 16S rRNA gene variability in different taxa, might have also played a part [94, 95]. Furthermore, previous studies have shown that *P. damselae* can be easily detected by cultivation method [96, 97], so it might have been preferentially cultivated and detected. However, the presence of *P. damselae*, regardless of the subspecies, could pose a risk for aquaculture facilities in the Adriatic Sea. Both subspecies of *P. damselae* are among the most important fish pathogens causing disease in a wide range of fish hosts [19, 98], including GSB [99, 100], ESB [101] and meagre (*Argyrosomus regius*) [102], being associated with mass mortalities in aquaculture facilities [103]. Although the fish collected for this study showed no signs of infection either externally or in the internal organs, we speculate that a change in environmental factors, such as temperature, or the presence of other stressors could trigger a disease outbreak. In fact, outbreaks of disease caused by these pathogens have been recorded mainly during the summer season and early autumn, when sea temperatures rise [104]. It is known that higher temperatures induce transcriptional changes that facilitate the development of a sufficient bacterial population to cause disease. This includes the upregulation of genes involved in DNA synthesis, nutrient uptake, chemotaxis, flagellar motility, secretion systems and antimicrobial resistance, as well as several plasmid-encoded virulence factors [105]. Nevertheless, cases of pseudotuberculosis have also been detected at low water temperatures [106]. This seasonality is probably also a contributing factor as to why a high proportion of the isolates belonged to *P. damselae*, as the fish were sampled in June and July. The prevalence observed here and the potential to cause disease outbreaks with the change in water temperature may also pose a risk to human health when handling the infected and diseased fish, as *P. damselae* subsp. *damselae* is known to cause severe disease in humans [107, 108].

The second most common genus was *Bacillus*, which accounted for almost a quarter of all cultured isolates. By the 16S rRNA gene amplicon sequencing, the presence of *Bacillus* was detected only in three samples – two GSB samples from farm location A and C in total of 0.03 and 40.86%, respectively, and in ESB sample from farm location D in total of 0.14%. The genus *Bacillus* includes many species that are ubiquitous in nature and have both medical and industrial importance due to their ability to secrete large amounts of enzymes and secondary metabolites. These include ribosomal peptides, volatile compounds, polyketides, non-ribosomal peptides, and hybrids between polyketides and non-ribosomal peptides [56]. Live cells or metabolites of several species of this genus, most of which belong to the *B. subtilis* group, have been shown to modulate the immune response of fish and increase disease resistance to many important bacterial fish pathogens in vitro, in vivo or in the gnotobiotic zebrafish model, including *A. hydrophila* [58, 109, 110], *E. tarda* [46], *P. damselae* subsp. *piscicida* [111], *Pseudomonas aeruginosa* [109], *Streptococcus iniae* [112, 113], *Streptococcus agalactiae* [114], *V. anguillarum* [37–39, 46], *V. alginolyticus* [109] and *Yersinia ruckeri* [115]. While the mechanisms by which different *Bacillus* spp. stimulate the immune response are not yet entirely clear, their increased disease resistance is mediated by several secondary metabolites with antibacterial and antifungal activity [56]. Therefore, it is possible that in certain samples a rather high number of isolates belonging to *Bacillus* and their secreted secondary metabolites are the cause for the low number or absence of *P. damselae* among the culturable isolates. Higher detection of genus *Bacillus* by 16S rRNA gene amplicon sequencing compared to *P. damselae*, especially on farm location C, supports this hypothesis.

On this basis, we selected a single *Bacillus* spp. isolate to characterise its biosynthetic and immunostimulatory potential. According to the Genome Taxonomy Database, the isolate was identified as *B. velezensis*, while antiSMASH analysis identified eight biosynthetic gene clusters (BGCs) for the common secondary metabolites of *B. velezensis*, i.e. surfactin, bacillaene, macrolactin, difficidin, bacillibactin, bacilysin, fengycin and iturin. Surfactins, fengycins and iturins are non-ribosomal lipopeptides produced by the *B. subtilis* group [56]. Fengycins and iturins have been shown to have potent antifungal activity against various phytopathogenic fungi and those that can cause disease in humans [116–118]. Surfactins, on the other hand, are potent broad-spectrum antibiotics that are effective against mycoplasmas, enveloped viruses and various bacteria, regardless of their Gram stain, possibly due to their pore-forming effect on the lipid membrane [119]. Bacilysin, another non-ribosomal peptide, also shows strong antibacterial activity by inhibiting glucosamine-6-phosphate synthase, which is necessary for the biosynthesis of peptidoglycans as components of the bacterial cell wall [120]. Similar to surfactin, the polyketides bacillaene, difficidin (and its derivative oxydifficidin) and various macrolactins exhibit potent antibacterial activity against many plant- and clinically important pathogens, including vancomycin- and methicillin-resistant isolates [121–125]. Finally, bacillibactin is a catecholic siderophore that chelates iron and reduces its bioavailability [126]. We hypothesise that some of these secondary metabolites are also effective against bacteria that are pathogenic to ESB and GSB, particularly emerging bacteria such as *V. harveyi*. Touraki et al. [111] have shown that a 16 kDa lipopeptide bacteriocin from *B. subtilis* inhibits the growth of two important fish pathogens, *V. anguillarum* and *P. damselae* subsp. *piscicida*. Although the production of these secondary metabolites was not tested in this *B. velezensis* isolate, the in silico biosynthetic potential indicated by the genome analysis and the results of a preliminary in vitro test suggesting bacteriostatic activity against *P. damselae* subsp. *piscicida* (Bulfon and Volpatti, personal communication) indicate that some of these metabolites are indeed secreted. However, their production and secretion in an aquatic environment, especially in a harsh gut environment, requires further investigation as the production of surfactin, iturin and fengycin depends on the temperature and aerobic conditions in the environment [127]. Future studies on aquaculture isolates of *B. velezensis* should therefore focus on confirming the synthesis of these metabolites in different cultivation environments and testing the activity of the purified metabolites against a range of fish pathogenic bacteria, especially those that are still exclusively controlled with antibiotics.

In contrast to the extensive research on this bacterium as a pest control agent against many plant pathogens in agriculture (reviewed in [128–131]), there are few studies on *B. velezensis* from aquatic environments. The BGCs identified here are consistent with recently published genome mining studies of two *B. velezensis* isolates from aquatic animals, the giant freshwater shrimp (*Macrobrachium rosenbergii*) [132] and the ribbontail (*Taeniura lymna*) and Tahitian stingrays (*Himantura fai*). In addition, the genome of *B. velezensis* also contains glycosidase hydrolase family of enzymes and the addition of raffinose and inulin to the culture medium significantly increases the growth of *B. velezensis* cultures [133], suggesting that dietary inclusion of these prebiotics with *B. velezensis* likely has a synergistic effect on host health. Such a synergistic effect would be beneficial from a disease prevention and control perspective in aquaculture, as different isolates of *B. velezensis* have shown in vitro broad-spectrum antimicrobial activity against various fish pathogens belonging to the genera *Aeromonas*, *Edwarsiella*, *Lactococcus*, *Streptococcus* and *Vibrio* [134–137]. In addition, dietary inclusion of this bacterium significantly increased disease resistance to *V. anguillarum*, *S. agalactiae*, *A. hydrophila* and *V. harveyi* in ESB [37, 38], Nile tilapia (*Oreochromis niloticus*) [134], *Carassius auratus* [135] and hybrid groupers (*Epinephelus lanceolatus* ♂ × *E. fuscoguttatus* ♀) [137].

Although probiotics are widely used for their beneficial effects, a safety assessment of a potential probiotic strain is required as they can express virulence factors or acquire antibiotic resistance genes. We have shown that the present isolate of *B. velezensis* is sensitive to all eight antibiotics recommended by EFSA [67], in addition to imipenem and meropenem as recommended by EUCAST [66], and is therefore currently considered safe for use as a fish feed additive.

To test the immunostimulatory potential of the selected *B. velezensis* isolate, we stimulated fish peripheral blood leukocytes (PBL) from ESB with live bacterial cells and lipoteichoic acid as a positive control for 3, 6 and 18 h. Although probiotics are usually administered orally with food and therefore react first with the gut-associated lymphoid tissue (GALT), we decided in favour of the PBL in vitro assay for a number of reasons. Firstly, probiotics not only have a direct effect on mucosal immunity, but can also modulate systemic immunity [28, 138]. Secondly, although we have not investigated the composition of immune cells in PBLs, it has already been shown that PBLs prepared by hypotonic lysis of erythrocytes contain the major cell types such as monocytes/macrophages, neutrophils and T and B cells, like peripheral blood mononuclear cells (PBMCs) prepared by gradient centrifugation. In addition, PBLs prepared in this way can respond to stimulation by pathogen-associated molecular patterns (PAMPs) and cytokines, proliferate and phagocytose [139]. Finally, working with PBLs is in line with the 3Rs principle (replace, reduce, refine) as peripheral blood is easy to obtain without the need to sacrifice the animals, as is the case with GALT.

A strong and transient pro-inflammatory response was observed in both *B. velezensis*- and lipoteichoic acid (LTA)-stimulated PBLs, supported by the sudden expression of the three pro-inflammatory cytokines *tnfa*, *il1b* and *il6*. Among these, *il1b* has generally been the most strongly expressed, but all targets gradually decreased after six hours. All three cytokines are master inducers of inflammation and enhance the antimicrobial functions of the immune cells, which facilitates the elimination of the pathogen [140, 141]. TNFα is a central inflammatory mediator with pleiotropic functions that is expressed by macrophages in the early phase of an infection. Like its counterpart in mammals, TNFα in teleosts promotes chemotaxis of neutrophils and monocytes/macrophages to the site of infection, phagocytosis of macrophages and triggers the production of reactive oxygen and nitrogen intermediates [142–144]. Considering that macrophages are the primary source of TNFα [145], the high log2 fold change (log2FC) observed for this gene suggests successful priming of ESB monocytes/macrophages from peripheral blood by both live bacteria and LTA. In addition, TNFα enhances the expression of proinflammatory cytokines, including *il1b* and *il6*, through NF-κB signalling [146, 147]. Indeed, we observed a very high expression (log2FC > 6) of *il1b* 3 and 6 h after stimulation with both live bacteria and LTA. Although the expression of this cytokine is also triggered by NF-κB signalling through the binding of the pathogen to surface receptors (e.g. toll-like receptors, TLRs) [148], this stimulatory effect of TNFα could partly explain the very high log2FC observed for *il1b*. This cytokine has multiple biological functions that overlap with those of TNFα [140], but unlike its mammalian counterpart it doesn’t have pyrogenic effect in teleosts [149]. In addition to triggering an inflammatory reaction, IL-1β activates T and B cells, natural killer cells, stimulates macrophages to produce inflammatory mediators and enhances phagocytic activity of phagocytes [150]. Although *il1b* is usually not constitutively expressed in teleosts, it is rapidly and strongly induced after stimulation by PAMPs such as lipopolysaccharide (LPS) or bacterial DNA [149–153]. Therefore, the strong induction of this cytokine that we observed here further confirms successful priming with applied stimuli, even though in mammals, adherence of PBMC to glass or polystyrene culture dishes can also trigger *il1b* expression [154]. So, this culturing effect may be partially responsible for the increased expression of *il1b* in the unstimulated controls at 18 h. IL-6 is the most pleiotropic proinflammatory cytokine and a key cytokine of the acute phase response. In contrast to *il1b*, *il6* is constitutively expressed in various fish tissues such as muscle, skin, spleen, head and trunk kidney and PBLs, although the level of expression in these tissues varies between species [155–157]. Similarly to the previous two cytokines, its expression is strongly upregulated in different tissues following bacterial (LPS, DNA) or viral (poly I:C) stimulation [156–159]. In teleosts, IL-6 has been shown to promote the differentiation of naïve T helper cells into Th2 cells [158], antibody production via the STAT3 signalling pathway [155] and to regulate function of monocytes/macrophages to secrete proinflammatory cytokines as well as their phagocytic and bactericidal ability [159]. Taken together, the expression of these cytokines indicates a strong innate immune response that probably triggers an adaptive response after prolonged stimulation. Nevertheless, excessive production of proinflammatory cytokines can be harmful and lead to immunopathologies. Therefore, their expression must be carefully balanced, either by transcriptional silencing or by secretion of anti-inflammatory mediators such as IL-10 or transforming growth factor β (TGF-β). This agrees well with the observed strong induction of *il10* after stimulation with both *B. velezensis* and LTA, which was triggered as early as 3 h after stimulation, probably to compensate for the sudden inflammatory response triggered by the stimuli. This indicates a beneficial regulation of the immune response rather than uncontrolled inflammation, suggesting that the tested probiotic candidate is an immune modulator, rather than only a stimulator of inflammatory response.

While the expression of pro-inflammatory cytokines following bacterial stimulation, as observed here, is to be expected, the lack or low induction of *tlr2* is somewhat puzzling. This is because TLR2 is one of the pattern recognition receptors (PRRs) responsible for the recognition of LTA and peptidoglycan (PGN) as cell wall components of Gram-positive bacteria, both in mammals and teleosts [148, 160], and the subsequent induction of pro-inflammatory cytokines via the Myd88-dependent signalling pathway [148]. Since we did not use specific pathogen-free (SPF) animals in this study, it is possible that the basal expression of *tlr2* in the experimental animals was already high enough due to previous exposure to different bacteria that could have triggered an immune response upon binding of a ligand, i.e. *B. velezensis* or LTA. Indeed, we detected a downregulation of *tlr2* almost throughout the experiment compared to the non-stimulated controls. While such expression profile of *tlr2* was not influenced by the immune status of the fish used in the immunostimulatory experiment, as the animals expectedly expressed other immune targets, we hypothesised that downregulation of *tlr2* indicates either its transcriptional or post-transcriptional repression in response to the inflammatory response, in addition to *il10* expression. Considering the redundancy of teleost genomes and the much higher number of TLRs identified in teleosts compared to mammals [148], other receptors might be involved in LTA or PGN recognition. Indeed, in yellow catfish (*Pelteobagrus fulvidraco*), TLR18 expression was found to be increased after stimulation of leukocytes with TLR ligands, including peptidoglycan [161]. In previous studies with *B. velezensis*, this probiotic was usually administered with the feed and the humoral and innate immune responses, particularly in the head kidney leukocytes (HKL), were observed. In tilapia, feeding *B. velezensis* resulted in a significant increase in lysozyme and superoxide dismutase activity, as well as significant upregulation of type C lysozyme, complement C3 and major histocompatibility complex class IIβ in the intestine, gills and head kidney [134]. Similarly, in ESB fed *B. velezensis*, both lysozyme and nitric oxide were significantly increased in serum, along with a marked upregulation of pro-inflammatory genes (*il1b*, *tnfa*, *cox2*) and antimicrobial peptides (dicentracin) in HKL [38]. Moreover, both *il1b* and *tnfa* were upregulated in *Carassius auratus* after dietary administration of this bacterium, in addition to an increased innate humoral response, i.e. serum acid and alkaline phosphatase, glutathione peroxidase and lysozyme [135]. In contrast to the administration of live bacteria, which triggered a strong upregulation of innate immune system genes, treatment of seabream HKL with extracellular products of *B. velezensis* did not result in the increased expression of innate immune system genes. However, when these HKL were challenged with *E. tarda*, significant expression of *il6* was detected [46], suggesting that even extracellular molecules secreted by this bacterium can modulate the immune response of the fish. However, to fully characterise the protective role of *B. velezensis* in farmed fish, a follow up study should focus on in vivo trial with the isolate-enriched feed and subsequent challenge with a bacterial pathogen. While this would emanate more accurately the complex gut-associated immune response, it would necessitate a preliminary in vitro data as obtained herein. Although comparison of the results is complicated by the fact that, as Mladineo et al. [4] noted, there are probably no two studies using the same experimental design, our results are consistent with previous studies showing that *B. velezensis* elicits a distinct innate immune response in fish that likely transitions into an adaptive response after prolonged stimulation.

## Conclusions

In the present study, we showed that European seabass (ESB) and gilthead seabream (GSB) have a similar composition and diversity in their gut microbiota, as both species displayed comparable alpha diversity indices and a uniform microbial community structure. *Pseudomondota*, *Bacillota* and *Actinomycetota* were identified as the dominant phyla in the gut of both ESB and GSB. Analysis of culturable isolates revealed that *P. damselae* was the most abundant bacterial isolate in the intestines of ESB and GSB, followed by *Bacillus* spp., *Vibrio* spp., *Staphylococcus* spp. and *Psychrobacter* spp. A single *Bacillus* isolate was tested as a potential probiotic, identified as *B. velezensis* by whole genome sequencing. In addition, genome analysis revealed the presence of biosynthetic gene clusters for most of the common *B. velezensis* secondary metabolites, including surfactin, bacillaene and fengycin. Antibiotic susceptibility testing showed that *B. velezensis* was sensitive to several antibiotics, emphasising its potential as a safe probiotic for aquaculture applications. Stimulation of peripheral blood leukocytes with *B. velezensis* elicited a strong pro-inflammatory response, indicating its immunomodulatory potential. Differential expression of target genes (*il1b*, *il6, tnfa* and *il10*) was observed over time, suggesting dynamic immune regulation in response to bacterial stimulation. Based on our results, *B. velezensis* is a promising probiotic candidate for Mediterranean aquaculture The limitation of the study, however, is that it did not entail testing of the influence of different sampling and extraction methods, and inclusion of individual samples from a given population to account for inter-individual variability, instead of samples pooling. However, to get a deeper insight into the intestinal microbiota composition, which can serve as a basis for studying microbiota dysbiosis in different diseases or resulting from introduction of novel feed formulations, a larger comprehensive study is needed. In addition, conducting metagenomic studies in future, rather than intestinal metabarcoding will provide more robust insight into the functional potential of intestinal microbiota, helping in the prevention of disease caused by emerging and re-emerging bacterial pathogens in Mediterranean aquaculture.

## Supplementary Information

Additional file 1: Sample IDs with Total Read Counts for each sample

Additional file 2: Rarefaction curve of number of read counts

Additional file 3: Table of contrasts generated in ‘limma’ package used to compare each treatment against its time-matched control, differences between treatments and time points

Additional file 4: List of values for the three alpha diversity indices for each sample

Additional file 5: Heatmap of core microbiome at Phylum level. Scale represents prevalence

Additional file 6: Heatmap OTUs of core microbiota with p-values

Additional file 7: List of genetically identified cultured isolates from intestines of European seabass and gilthead seabream with counts per host species per farm and number of isolates belonging to different genera/species

## Data Availability

Raw sequence reads from intestinal microbiota metabarcoding were deposited in European Nucleotide Archive (ENA) (https://www.ebi.ac.uk/ena) under project accession number PRJEB72876. The genome assembly of Bacillus velezensis has been deposited in GenBank under BioProject accession number PRJNA1196159. *B. velezensis* isolate is deposited in the Laboratory of Aquaculture, Institute of Oceanography and Fisheries and will be made available on request.
