# Supplementary material for: Prospecting microbiota of Adriatic fish: Bacillus velezensis as a potential probiotic candidate
Source: Anim Microbiome. 2025 Jun 14;7:64. doi: 10.1186/s42523-025-00429-5 (PMC12167591; doi:10.1186/s42523-025-00429-5)
Supplement: Supplementary file 1 — Additional file 1: Sample IDs with Total Read Counts for each sample [file 42523_2025_429_MOESM1_ESM.docx]

**Supplementary Table 1.** Sample IDs with Total Read Counts for each sample. Fish farm location (A – H) match the labels on Figure 1.

| **No.** | **Sample ID** | **Sample label** | **Species** | **Fish farm label** | **Total Read Counts QIIME2** | **Total Read Counts**  **R 'phyloseq' taxonomy filter** |
| --- | --- | --- | --- | --- | --- | --- |
| 1 | BDL | sample.1 | *Dicentrarchus labrax* | G | 68094 | 68056 |
| 2 | PDL | sample.10 | *Dicentrarchus labrax* | H | 57850 | 57840 |
| 3 | PSA | sample.11 | *Sparus aurata* | H | 71432 | 71432 |
| 4 | SDL | sample.12 | *Dicentrarchus labrax* | A | 71226 | 42295 |
| 5 | SSA | sample.13 | *Sparus aurata* | A | 85622 | 85622 |
| 6 | VDL | sample.14 | *Dicentrarchus labrax* | D | 67689 | 67689 |
| 7 | VSA | sample.15 | *Sparus aurata* | D | 66244 | 66244 |
| 8 | FDL | sample.2 | *Dicentrarchus labrax* | C | 57035 | 57030 |
| 9 | FSA | sample.3 | *Sparus aurata* | C | 45124 | 45124 |
| 10 | KDL | sample.4 | *Dicentrarchus labrax* | E | 72645 | 53852 |
| 11 | KSA | sample.5 | *Sparus aurata* | E | 84951 | 84892 |
| 12 | LDL | sample.6 | *Dicentrarchus labrax* | F | 72231 | 72221 |
| 13 | LSA | sample.7 | *Sparus aurata* | F | 56006 | 42452 |
| 14 | ODL | sample.8 | *Dicentrarchus labrax* | B | 72913 | 72913 |
| 15 | OSA | sample.9 | *Sparus aurata* | B | 61824 | 61824 |
| **Total read counts:** | | | | | 1,010,886 | 949,486 |
