# Supplementary figures and images for: Prospecting microbiota of Adriatic fish: Bacillus velezensis as a potential probiotic candidate
Source: Anim Microbiome. 2025 Jun 14;7:64. doi: 10.1186/s42523-025-00429-5 (PMC12167591; doi:10.1186/s42523-025-00429-5)

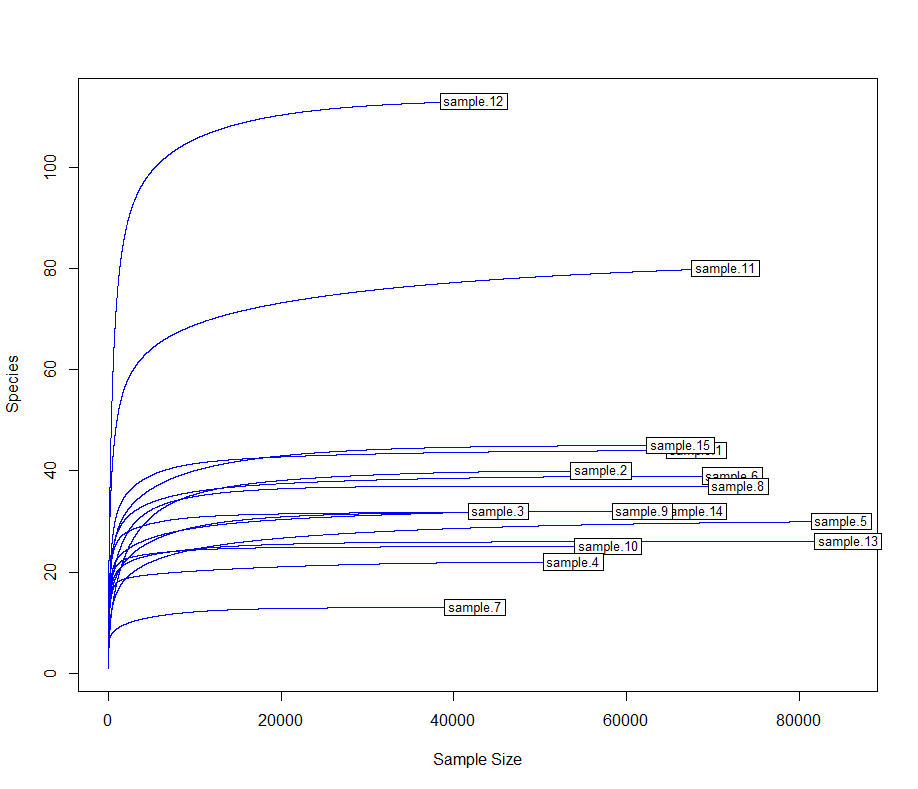

Supplement: Supplementary file 2 — Additional file 2: Rarefaction curve of number of read counts [file 42523_2025_429_MOESM2_ESM.tiff]
