# Supplementary material for: Prospecting microbiota of Adriatic fish: Bacillus velezensis as a potential probiotic candidate
Source: Anim Microbiome. 2025 Jun 14;7:64. doi: 10.1186/s42523-025-00429-5 (PMC12167591; doi:10.1186/s42523-025-00429-5)
Supplement: Supplementary file 4 — Additional file 4: List of values for the three alpha diversity indices for each sample [file 42523_2025_429_MOESM4_ESM.docx]

**Supplementary Table 2.** List of values for the three alpha diversity indices for each sample. Fish farm location (A – H) match the labels on Fig. 1.

| **No.** | **Sample ID** | **Sample label** | **Species** | **Fish farm label** | **Observed ASV** | **Pielou** | **Shannon** |
| --- | --- | --- | --- | --- | --- | --- | --- |
| 1 | BDL | sample.1 | *Dicentrarchus labrax* | G | 44 | 0,56 | 2,11 |
| 2 | PDL | sample.10 | *Dicentrarchus labrax* | H | 25 | 0,66 | 2,14 |
| 3 | PSA | sample.11 | *Sparus aurata* | H | 75 | 0,69 | 2,97 |
| 4 | SDL | sample.12 | *Dicentrarchus labrax* | A | 113 | 0,55 | 2,62 |
| 5 | SSA | sample.13 | *Sparus aurata* | A | 26 | 0,61 | 1,98 |
| 6 | VDL | sample.14 | *Dicentrarchus labrax* | D | 32 | 0,52 | 1,80 |
| 7 | VSA | sample.15 | *Sparus aurata* | D | 45 | 0,50 | 1,89 |
| 8 | FDL | sample.2 | *Dicentrarchus labrax* | C | 40 | 0,11 | 0,40 |
| 9 | FSA | sample.3 | *Sparus aurata* | C | 32 | 0,66 | 2,30 |
| 10 | KDL | sample.4 | *Dicentrarchus labrax* | E | 22 | 0,63 | 1,96 |
| 11 | KSA | sample.5 | *Sparus aurata* | E | 28 | 0,54 | 1,79 |
| 12 | LDL | sample.6 | *Dicentrarchus labrax* | F | 39 | 0,64 | 2,35 |
| 13 | LSA | sample.7 | *Sparus aurata* | F | 13 | 0,54 | 1,37 |
| 14 | ODL | sample.8 | *Dicentrarchus labrax* | B | 37 | 0,50 | 1,81 |
| 15 | OSA | sample.9 | *Sparus aurata* | B | 32 | 0,68 | 2,36 |
