# Supplementary material for: Prospecting microbiota of Adriatic fish: Bacillus velezensis as a potential probiotic candidate
Source: Anim Microbiome. 2025 Jun 14;7:64. doi: 10.1186/s42523-025-00429-5 (PMC12167591; doi:10.1186/s42523-025-00429-5)
Supplement: Supplementary file 5 — Additional file 5: Heatmap of core microbiome at Phylum level. Scale represents prevalence [file 42523_2025_429_MOESM5_ESM.pdf]

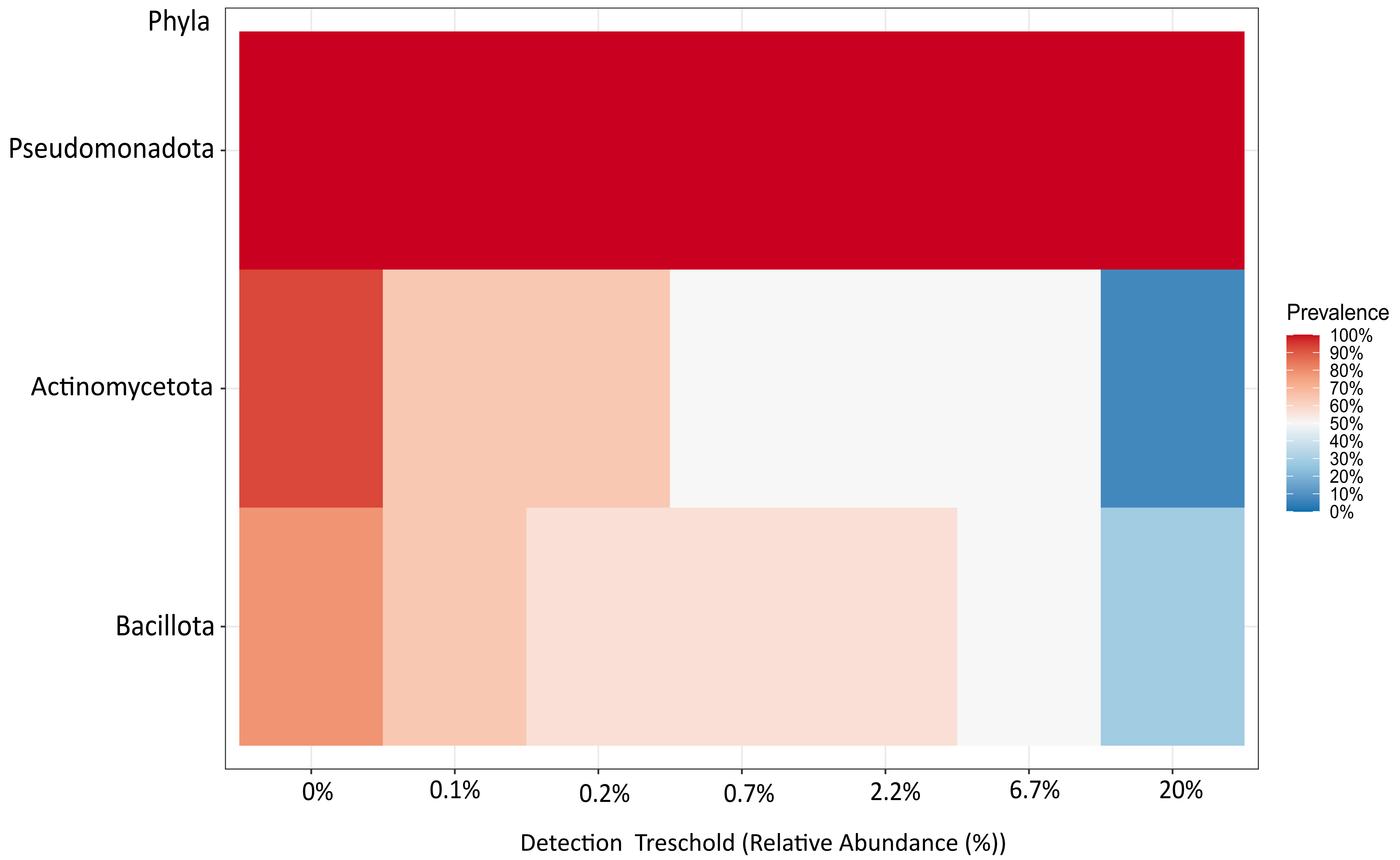

Phyla
Pseudomonadota
Actinomycetota
Bacillota
0%
0.1%
0.2%
0.7%
2.2%
6.7%
20%
Detection Treschold (Relative Abundance (%))
Prevalence
100%
90%
80%
70%
60%
50%
40%
30%
20%
10%
0%
