# Supplementary material for: Prospecting microbiota of Adriatic fish: Bacillus velezensis as a potential probiotic candidate
Source: Anim Microbiome. 2025 Jun 14;7:64. doi: 10.1186/s42523-025-00429-5 (PMC12167591; doi:10.1186/s42523-025-00429-5)
Supplement: Supplementary file 7 — Additional file 7: List of genetically identified cultured isolates from intestines of European seabass and gilthead seabream with counts per host species per farm and number of isolates belonging to different genera/species [file 42523_2025_429_MOESM7_ESM.docx]

**Supplementary Table 3. List of genetically identified cultured bacterial isolates form intestines of European sea bass and Gilthead seabream.**

| **Host/location** | **Sample ID** | **Genus / species** |  |
| --- | --- | --- | --- |
| *Dicentrarchus labrax*  Farm A (Brač Island) | DL_A1 | *Kocuria* |  |
|  | DL_A2 | *Bacillus* |  |
|  | DL_A3 | *Brevundimonas bullata* |  |
|  | DL_A4 | *Bacillus* |  |
|  | DL_A5 | *Glutamicibacter* |  |
|  | DL_A6 | *Pseudoclavibacter* |  |
|  | DL_A7 | *Pseudochrobactrum* |  |
|  | DL_A8 | *Bacillus* |  |
|  | DL_A9 | *Bacillus* |  |
|  | DL_A10 | *Kocuria* |  |
| *Sparus aurata*  Farm A (Brač Island) | SA_A11 | *Photobacterium damselae* |  |
|  | SA_A12 | *Photobacterium damselae* |  |
|  | SA_A13 | *Staphylococcus* |  |
|  | SA_A14 | *Photobacterium damselae* |  |
|  | SA_A15 | *Photobacterium damselae* |  |
|  | SA_A16 | *Staphylococcus* |  |
|  | SA_A17 | *Staphylococcus* |  |
|  | SA_A18 | *Photobacterium damselae* |  |
|  | SA_A19 | *Staphylococcus* |  |
|  | SA_A20 | *Photobacterium damselae* |  |
| *Dicentrarchus labrax*  Farm B (Cres Island) | DL_B1 | Not identified |  |
|  | DL_B2 | *Kocuria* |  |
|  | DL_B3 | *Psychrobacter* |  |
|  | DL_B4 | *Paracoccus* |  |
|  | DL_B5 | *Psychrobacter* |  |
|  | DL_B6 | *Psychrobacter* |  |
|  | DL_B7 | *Dietzia* |  |
|  | DL_B8 | *Psychrobacter* |  |
|  | DL_B9 | *Cognatiyoonia* |  |
|  | DL_B10 | *Psychrobacter* |  |
| *Sparus aurata*  Farm B (Cres Island) | SA_B11 | *Rhodococcus* |  |
|  | SA_B12 | Not identified |  |
|  | SA_B13 | *Rhodococcus* |  |
|  | SA_B14 | *Rhodococcus* |  |
|  | SA_B15 | *Plantibacter* |  |
|  | SA_B16 | *Sphingobacterium spiritivorum* |  |
|  | SA_B17 | *Stenotrophomonas rhizophila* |  |
|  | SA_B18 | *Stenotrophomonas rhizophila* |  |
|  | SA_B19 | *Stenotrophomonas rhizophila* |  |
|  | SA_B20 | *Psychrobacter* |  |

**Supplementary Table 3. continued**

| **Host/location** | **Sample ID** | **Genus / species** |  |
| --- | --- | --- | --- |
| *Dicentrarchus labrax*  Farm C (Movar Bay) | DL_C1 | *Bacillus* |  |
|  | DL_C2 | *Vibrio* |  |
|  | DL_C3 | *Vibrio* |  |
|  | DL_C4 | Not identified |  |
|  | DL_C5 | *Vibrio* |  |
|  | DL_C6 | *Photobacterium damselae* |  |
|  | DL_C7 | *Vibrio* |  |
|  | DL_C8 | *Vibrio* |  |
|  | DL_C9 | *Vibrio* |  |
|  | DL_C10 | *Vibrio* |  |
| *Sparus aurata*  Farm C (Movar Bay) | SA_C11 | *Photobacterium damselae* |  |
|  | SA_C12 | Not identified |  |
|  | SA_C13 | *Photobacterium damselae* |  |
|  | SA_C14 | *Vibrio* |  |
|  | SA_C15 | *Vibrio* |  |
|  | SA_C16 | *Photobacterium damselae* |  |
|  | SA_C17 | *Photobacterium damselae* |  |
|  | SA_C18 | *Photobacterium damselae* |  |
|  | SA_C19 | *Photobacterium damselae* |  |
|  | SA_C20 | *Photobacterium damselae* |  |
| *Dicentrarchus labrax*  Farm D (Rava channel) | DL_D1 | *Photobacterium damselae* |  |
|  | DL_D2 | *Vibrio* |  |
|  | DL_D3 | *Pseudomonas* |  |
|  | DL_D4 | *Vibrio* |  |
|  | DL_D5 | *Vibrio* |  |
|  | DL_D6 | *Vibrio* |  |
|  | DL_D7 | *Photobacterium damselae* |  |
|  | DL_D8 | *Photobacterium damselae* |  |
|  | DL_D9 | *Vibrio* |  |
|  | DL_D10 | Not identified |  |
| *Sparus aurata*  Farm D (Rava channel) | SA_D11 | *Shewanella putrefaciens* |  |
|  | SA_D12 | *Bacillus* |  |
|  | SA_D13 | *Pseudomonas* |  |
|  | SA_D14 | *Bacillus* |  |
|  | SA_D15 | *Sporosarcina* |  |
|  | SA_D16 | *Bacillus* |  |
|  | SA_D17 | *Shewanella putrefaciens* |  |
|  | SA_D18 | *Aeromonas* |  |
|  | SA_D19 | *Pseudomonas* |  |
|  | SA_D20 | *Bacillus* |  |

**Supplementary Table 3. continued**

| **Host/location** | **Sample ID** | **Genus / species** |  |
| --- | --- | --- | --- |
| *Sparus aurata*  Farm E (Košara Island) | SA_E1 | *Bacillus* |  |
|  | SA_E2 | *Bacillus* |  |
|  | SA_E3 | *Bacillus* |  |
|  | SA_E4 | *Bacillus* |  |
|  | SA_E5 | *Bacillus* |  |
|  | SA_E6 | *Bacillus* |  |
|  | SA_E7 | *Bacillus* |  |
|  | SA_E8 | *Bacillus* |  |
|  | SA_E9 | *Bacillus* |  |
|  | SA_E10 | *Bacillus* |  |
| *Dicentrarchus labrax*  Farm E (Košara Island) | DL_E11 | *Photobacterium damselae* |  |
|  | DL_E12 | *Photobacterium damselae* |  |
|  | DL_E13 | *Photobacterium damselae* |  |
|  | DL_E14 | *Photobacterium damselae* |  |
|  | DL_E15 | *Photobacterium damselae* |  |
|  | DL_E16 | *Photobacterium damselae* |  |
|  | DL_E17 | *Photobacterium damselae* |  |
|  | DL_E18 | *Photobacterium damselae* |  |
|  | DL_E19 | *Photobacterium damselae* |  |
|  | DL_E20 | *Photobacterium damselae* |  |
| *Dicentrarchus labrax*  Farm F (Lamjana, Ugljan Island) | DL_F1 | Pseudolateromonas |  |
|  | DL_F2 | *Photobacterium damselae* |  |
|  | DL_F3 | *Photobacterium damselae* |  |
|  | DL_F4 | Not identified |  |
|  | DL_F5 | *Photobacterium damselae* |  |
|  | DL_F6 | *Photobacterium damselae* |  |
|  | DL_F7 | *Vibrio* |  |
|  | DL_F8 | *Acinetobacter johnsonii* |  |
|  | DL_F9 | *Photobacterium damselae* |  |
|  | DL_F10 | *Staphylococcus* |  |
| *Sparus aurata*  Farm F (Lamjana, Uhljan Island) | SA_F11 | *Staphylococcus* |  |
|  | SA_F12 | *Staphylococcus* |  |
|  | SA_F13 | *Staphylococcus* |  |
|  | SA_F14 | *Photobacterium damselae* |  |
|  | SA_F15 | *Photobacterium damselae* |  |
|  | SA_F16 | *Staphylococcus* |  |
|  | SA_F17 | *Staphylococcus* |  |
|  | SA_F18 | *Photobacterium damselae* |  |
|  | SA_F19 | *Staphylococcus* |  |
|  | SA_F20 | *Staphylococcus* |  |

**Supplementary Table 3. continued**

| **Host/location** | **Sample ID** | **Genus / species** |  |
| --- | --- | --- | --- |
| *Dicentrarchus labrax*  Farm G (Budava, Istri) | DL_G1 | *Vibrio* |  |
|  | DL_G2 | *Vibrio* |  |
|  | DL_G3 | Not identified |  |
|  | DL_G4 | *Vibrio* |  |
|  | DL_G5 | *Photobacterium damselae* |  |
|  | DL_G6 | *Photobacterium damselae* |  |
|  | DL_G7 | *Photobacterium damselae* |  |
|  | DL_G8 | *Photobacterium damselae* |  |
|  | DL_G9 | *Vibrio* |  |
|  | DL_G10 | *Photobacterium aphoticum* |  |
| *Sparus aurata*  Farm H (Pelješac peninsula) | SA_H1 | *Bacillus* |  |
|  | SA_H2 | *Bacillus* |  |
|  | SA_H3 | *Bacillus* |  |
|  | SA_H4 | *Bacillus* |  |
|  | SA_H5 | *Bacillus* |  |
|  | SA_H6 | *Bacillus* |  |
|  | SA_H7 | *Bacillus* |  |
|  | SA_H8 | *Bacillus* |  |
|  | SA_H9 | *Bacillus* |  |
|  | SA_H10 | *Bacillus* |  |
| *Dicentrarchus labrax*  Farm H (Pelješac peninsula) | DL_H11 | *Bacillus* |  |
|  | DL_H12 | *Staphylococcus* |  |
|  | DL_H13 | *Bacillus* |  |
|  | DL_H14 | *Staphylococcus* |  |
|  | DL_H15 | *Bacillus* |  |
|  | DL_H16 | *Bacillus* |  |
|  | DL_H17 | *Staaphylococcus* |  |
|  | DL_H18 | *Photobacterium damselae* |  |
|  | DL_H19 | *Bacillus* |  |
|  | DL_H20 | *Photobacterium damselae* |  |

**Supplementary Table 4. Number of genera identified per host species per farm**

| **Host species** | **Location** | **Nr of genera / species** |
| --- | --- | --- |
| *Dicentrarchus labrax* | Farm A | 6 |
| *Sparus aurata* | Farm A | 2 |
| *Dicentrarchus labrax* | Farm B | 5 |
| *Sparus aurata* | Farm B | 5 |
| *Dicentrarchus labrax* | Farm C | 3 |
| *Sparus aurata* | Farm C | 2 |
| *Dicentrarchus labrax* | Farm D | 3 |
| *Sparus aurata* | Farm D | 5 |
| *Sparus aurata* | Farm E | 1 (exclusively *Bacillus*) |
| *Dicentrarchus labrax* | Farm E | 1 (exclusively *Photobacterium damselae*) |
| *Dicentrarchus labrax* | Farm F | 5 |
| *Sparus aurata* | Farm F | 2 |
| *Dicentrarchus labrax* | Farm G | 3 |
| *Sparus aurata* | Farm H | 1 (exclusively *Bacillus*) |
| *Dicentrarchus labrax* | Farm H | 3 |

**Supplementary Table 5. Counts of all genetically identified cultured isolate from intestines of European sea bass and gilthead seabream**

| **Genus / species** | **Nr of isolates** |
| --- | --- |
| *Photobacterium damselae* | 42 |
| *Bacillus* | 34 |
| *Vibrio* | 19 |
| *Staphylococcus* | 15 |
| *Psychrobacter* | 6 |
| *Kocuria* | 3 |
| *Pseudomonas* | 3 |
| *Rhodococcus* | 3 |
| *Stenotrophomonas rhizophila* | 3 |
| *Shewanella putrefaciens* | 2 |
| *Acinetobacter johnsonii* | 1 |
| *Aeromonas* | 1 |
| *Arthrobacter* | 1 |
| *Brevundimonas bullata* | 1 |
| *Cognatiyoonia* | 1 |
| *Dietzia* | 1 |
| *Paracoccus* | 1 |
| *Plantibacter* | 1 |
| *Pseudoalteromonas* | 1 |
| *Pseudochrobactrum* | 1 |
| *Pseudoclavibater* | 1 |
| *Spingobacterium spiritivorum* | 1 |
| *Sporosarcina* | 1 |
